# A Diazo Linker Ligand Promotes Flexibility and Induced Fit Binding in a Microporous Copper Coordination Network

**DOI:** 10.1002/anie.202507757

**Published:** 2025-06-10

**Authors:** Xia Li, Debobroto Sensharma, Wells Graham, Volodymyr Bon, En Lin, Xiang‐Jing Kong, Tao He, Andrey A. Bezrukov, Zhenjie Zhang, Stefan Kaskel, Timo Thonhauser, Michael J. Zaworotko

**Affiliations:** ^1^ College of Chemistry Nankai University Tianjin 300071 P.R.China; ^2^ Department of Chemical Science Bernal Institute University of Limerick Limerick V94 T9PX Republic of Ireland; ^3^ Department of Physics and Center for Functional Materials Wake Forest University Winston‐Salem North Carolina 27109 USA; ^4^ Faculty of Chemistry Technische Universität Dresden Bergstrasse 66 01062 Dresden Germany

**Keywords:** Azo bond, Flexible metal‐organic materials, Host‐guest chemistry, Induced fit

## Abstract

Flexible organic linkers represent an intuitive and effective strategy to design flexible metal–organic materials. We report herein a systematic study concerning the effect of varying the central bond of mixed pyridyl‐benzoate linkers, L, upon the flexibility of three isostructural **kdd** topology microporous coordination networks (CNs) of formula ML_2_: **X‐kdd‐1‐Cu**, **1** = L = (*E*)‐4‐(pyridin‐4‐yldiazenyl)benzoate; **X‐kdd‐2‐Cu**, **2** = L = (*E*)‐4‐(2‐(pyridin‐4‐yl)vinyl)benzoate; the previously reported **X‐kdd‐3‐Cu**, **3** = L = 4‐(pyridin‐4‐ylethynyl)benzoate. As revealed by single crystal x‐ray diffraction (SCXRD) and gas sorption studies, **X‐kdd‐1‐Cu**, exhibited gate‐opening during CO_2_ and hydrocarbon (C2 and C8) sorption experiments whereas the other two CNs did not. Insight into these phase transformations was gained from in situ variable‐pressure and variable temperature powder X‐ray diffraction (PXRD), SCXRD, and modeling. Rotation of ligand **1** around the diazo bond, torsion angle changes between phenyl and carboxylate moieties, and deformation of the Cu‐based rod building blocks enabled activated **X‐kdd‐1‐Cu** to form new phases with C8 isomers and CH_2_Cl_2_, CH_2_Cl_2_ inducing contraction of the activated phase. Computational studies suggest that **1** enables flexibility thanks to its lower barrier of deformation versus **2** or **3**. This study teaches that diazo moieties could offer a general strategy to enhance the flexibility of CNs.

## Graphical abstract

A family of three isostructural coordination networks differs only in the central part of the linker ligand. Whereas a linker comprising an azo bond enabled guest‐induced structural flexibility, including induced fit behavior, networks containing linkers with hydrocarbon moieties were observed to be rigid upon exposure to the same guests.

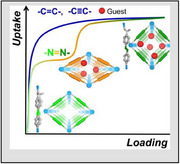

## Introduction

Metal−organic materials (MOMs)^[^ ^1^, ^2^ ^]^ such as metal–organic frameworks (MOFs)^[^ ^3^, ^4^ ^]^ and porous coordination polymers (PCPs)^[^ ^5^, ^6^ ^]^ are types of coordination networks (CNs) that offer potential utility to address the high‐energy footprint, costs, and risks associated with storage or purification of gases and vapors.^[^ ^7^, ^8^, ^9^, ^10^, ^11^, ^12^, ^13^, ^14^ ^]^ Whereas rigid microporous CNs typically display type I (Langmuir) isotherms, flexible MOMs (FMOMs)^[^ ^15^, ^16^, ^17^, ^18^, ^19^, ^20^, ^21^, ^22^ ^]^ can exhibit stepped isotherms that enhance working capacity in the context of pressure swing adsorption gas storage^[^ ^23^ ^]^ or selective guest inclusion for gas separation.^[^ ^24^ ^]^ Indeed, it is becoming increasingly clear that some flexible CNs can offer benchmark performance thanks to their ability to exhibit induced fit binding towards guest molecules.^[^ ^25^, ^26^, ^27^, ^28^, ^29^, ^30^, ^31^, ^32^ ^]^ Nevertheless, it remains a challenge to predict and design flexibility, especially the properties that might be imparted by flexibility. This study addresses linker ligand design in the context of FMOMs.

The mechanisms of flexibility in FMOMs can be classified into three categories^[^ ^33^, ^34^ ^]^: linker ligand induced (e.g., bending, twisting and/or rotation); metal node induced (e.g., deformation and/or reconstitution/isomerism); network displacement (e.g., interpenetrated or layered net sliding or expansion). The three mechanisms can work synergistically, although one tends to be dominant. With respect to the linker ligand, the use of a flexible organic linker is an intuitive strategy to prepare FMOMs.^[^ ^34^ ^]^ The relatively low rotation barrier of a single bond^[^ ^35^, ^36^ ^]^ means that organic linkers containing a moiety based upon a single bond (e.g., C─C, C─N bonds) can result in FMOMs. An early example of the effect of a C─C moiety was demonstrated by the structural diversity enabled by 4,4′‐dipyridylethane (bpe) in CNs versus analogs based upon 4,4‐bipyridine.^[^ ^37^ ^]^ Prototypal flexible CNs such as **ELM‐11** ^[^ ^38^, ^39^, ^40^, ^41^ ^]^ and **Cu_2_(bdc)_2_bipy**.^[^ ^42^, ^43^ ^]^ were driven by network displacement whereas **MIL‐53(Fe)** ^[^ ^44^, ^46^ ^]^ involved metal node distortion. Perhaps the first report of an FMOM involving a flexible linker concerned **Cu_2_(pzdc)_2_(dpyg)**, which comprises 4,4′dipridylglycol (dpyg) linkers.^[^ ^47^ ^]^ More recently, bpe was used to generate **sql‐SIFSIX‐bpe‐Zn**, a CN that exhibits induced fit binding and exceptionally strong binding for C_2_H_2_.^[^ ^27^ ^]^ Other examples of ligand‐induced flexibility are exemplified by **SIFSIX‐23‐Cu** ^[^ ^48^ ^]^ (a bisimidazolyl linker ligand) and **HNUST‐5** ^[^ ^49^ ^]^ (an acylamide moiety). Conversely, linker ligands with high rotation barriers, such as those with double bond moieties, tend not to enable flexibility with the exception of those comprising diazo moieties, as exemplified by our reports on **sql‐(azpy)(pdia)‐Ni** ^[^ ^50^ ^]^ and **X‐dia‐6‐Ni**.^[^ ^51^ ^]^

**Figure 1:**
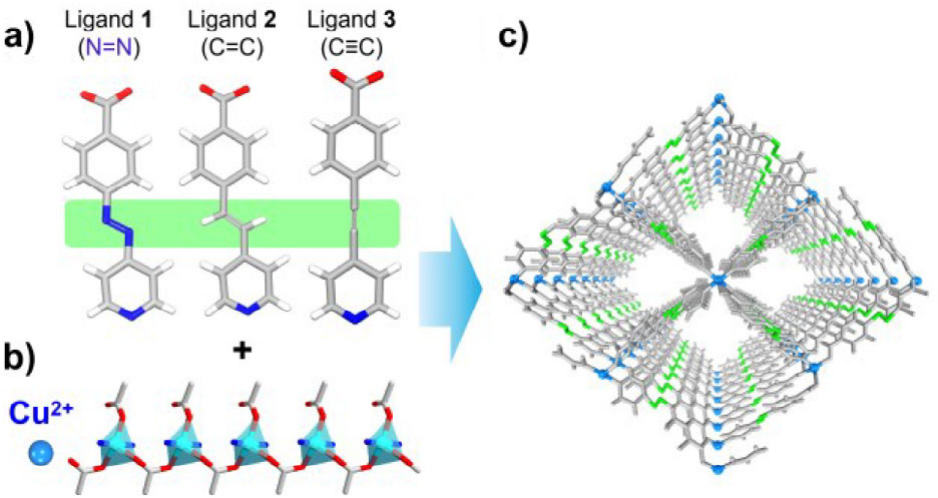
a) The ligands **1** = (*E*)‐4‐(pyridin‐4‐yldiazenyl) benzoate, **2** = (*E*)‐4‐(2‐(pyridin‐4‐yl) vinyl) benzoate, **3** = 4‐(pyridin‐4‐ylethynyl) benzoate. b) Cu‐based RBB. c) 3D Structure of **X‐kdd‐n‐Cu** (**n** = **1**–**3**) viewed along the crystallographic *a* axis.

Azo moieties are widely studied as photo‐responsive groups in organic linkers to study guest uptake modulated by irradiation.^[^ ^52^, ^53^, ^54^, ^55^ ^]^ *E‐Z* isomerization of the azo bond, which can be light^[^ ^56^ ^]^ or mechanically induced,^[^ ^57^ ^]^ drives such behavior. Overall, diazo moieties remain understudied as a source of structural flexibility without light stimulus in FMOMs. **sql‐(pdia)(azpy)‐Ni** ^[^ ^50^ ^]^ and **X‐dia‐6‐Ni**.^[^ ^51^ ^]^ exhibited significant deformation around the azo bond in pendent isophthalate and N/CO_2_ linkers, respectively, resulting in guest‐induced phase transformations. Nevertheless, there remains a dearth of systematic studies on diazo linkers. We address this matter herein through a crystal engineering study of a family of three CNs comprised of a Cu‐based rod building block (RBB) of general formula ML_2_ (L = a mixed pyridyl‐benzoate linker), one of which is the previously reported CN **X‐kdd‐3‐Cu**, Cu(peba)_2_,^[^ ^58^, ^59^ ^]^ **3** = 4‐(pyridin‐4‐ylethynyl) benzoate. That **X‐kdd‐3‐Cu** is amenable to fine‐tuning by ligand substitution prompted us to prepare and characterize **X‐kdd‐1‐Cu**, **1** = (*E*)‐4‐(pyridin‐4‐yldiazenyl) benzoate, and **X‐kdd‐2‐Cu**, **2** = (*E*)‐4‐(2‐(pyridin‐4‐yl) vinyl) benzoate (Figure 1). The parent for all three CNs is the **kdd** topology CN [Cu(C_6_H_4_O_2_N)_2_)·2H_2_O]_n_.^[^ ^60^ ^]^ A characteristic of this Cu RBB (Figure 1b) is that it precludes interpenetration and can enable high porosity by extending the length of the linker ligand. Our survey of the CSD revealed only 69 entries with this Cu RBB, of which 44 entries are 3D (most common topologies **fsc**, **kdd**, **hms**, **seh,** and **pcu**) and 25 entries are 2D (most common topologies **sql**, **hcb**, and **ins**), respectively (Version 5.44, April 2023, r S1). In this work, we focus on how the central bond (‐N═N‐, ‐C═C‐, and ‐C≡C‐) in the linker can impact structural flexibility through structural, sorption, and modelling studies.

## Results and Discussion

### Synthesis And Structural Analysis

**Figure 2:**
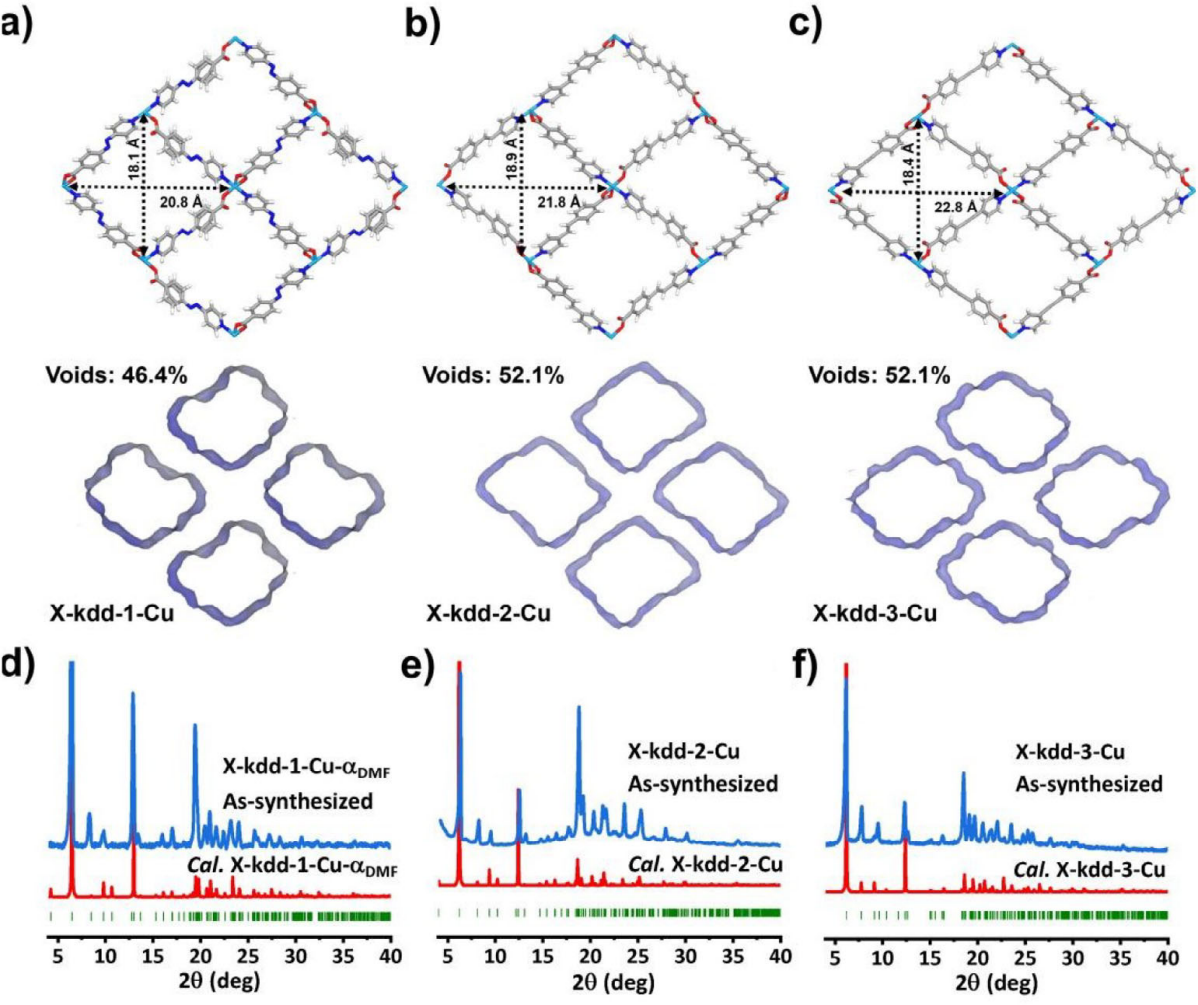
Pore size and calculated guest accessible volume (voids) of a) **X‐kdd‐1‐Cu**, b) **X‐kdd‐2‐Cu**, c) **X‐kdd‐3‐Cu**. Comparison of experimental PXRD patterns of d) **X‐kdd‐1‐Cu**, e) **X‐kdd‐2‐Cu,** and f) **X‐kdd‐3‐Cu,** and PXRD patterns calculated from the SCXRD determined crystal structures for **X‐kdd‐1‐Cu**, **X‐kdd‐3‐Cu,** and the PXRD determined crystal structure of **X‐kdd‐2‐Cu**.

Solvothermal reactions between the protonated ligands **1**–**3** (Figure 1a) and Cu^2+^ nitrate in *N, N*‐dimethylformamide (DMF) and ethanol (EtOH) afforded single crystals of **X‐kdd‐n‐Cu** (n = **1**, **3**) and a microcrystalline powder of **X‐kdd‐2‐Cu**. Single crystal X‐ray diffraction (SCXRD) revealed that **X‐kdd‐1‐Cu** and **X‐kdd‐3‐Cu**.^[^ ^58^, ^59^ ^]^ crystallized in the monoclinic space group *Cc* (Table S2). The powder X‐ray diffraction (PXRD) pattern of **X‐kdd‐2‐Cu** matches those of **X‐kdd‐1‐Cu** and **X‐kdd‐3‐Cu** (Figure S1) and was indexed and fitted using Pawley refinement. Subsequent refinement indicates that **X‐kdd‐2‐Cu** is isostructural with **X‐kdd‐1‐Cu** and **X‐kdd‐3‐Cu** (Figure S2 and Table S3). SCXRD studies and simulation experiments (see Supporting Information) indicate that all three CNs are comprised of Cu‐carboxylate RBBs (Figure 1b) cross‐linked by pyridyl carboxylate ligands (**1**, **2,** and **3**). The Cu^2+^ centre exhibits trigonal bipyramidal coordination geometry with two nitrogen (N) atoms and three carboxyl groups from five ligands. Linear ligands in **X‐kdd‐n‐Cu** (**n** = **1**, **2**, **3**) pack in face‐to‐face mode with their carboxyl groups or pyridine rings orienting in the same direction (Figure 1c). All three CNs exhibit 1D rectangular nanoscale pores parallel to the crystallographic *a*‐axis with diagonal distances between Cu cations of 20.8 × 18.1, 21.8 × 18.9 and 22.8 × 18.4Å for **X‐kdd‐1‐Cu**, **X‐kdd‐2‐Cu** and **X‐kdd‐3‐Cu**, respectively, and guest accessible volumes calculated by PLATON^[^ ^61^ ^]^ of 46.4%, 52.1%, and 52.1%, respectively (Figure 2a–c). The experimental PXRD patterns of **X‐kdd‐1‐Cu**, **X‐kdd‐2‐Cu,** and **X‐kdd‐3‐Cu** are consistent with those calculated from SCXRD data (Figure 2e,f). Thermogravimetric analysis (TGA) revealed that the as‐synthesized phases of **X‐kdd‐1‐Cu**, **X‐kdd‐2‐Cu,** and **X‐kdd‐3‐Cu** lose guest molecules at ∼130 °C with no further weight losses until ∼260 °C (Figure S3). Fourier Transform Infrared (FTIR) studies of as‐synthesized samples of **X‐kdd‐1‐Cu**, **X‐kdd‐2‐Cu,** and **X‐kdd‐3‐Cu** support the presence of guest DMF molecules in their pores as indicated by characteristic C═O stretching peaks of DMF at 1667_,_ 1661, and 1663 cm^−1^, respectively (Figure S4).

### Activation

**Figure 3:**
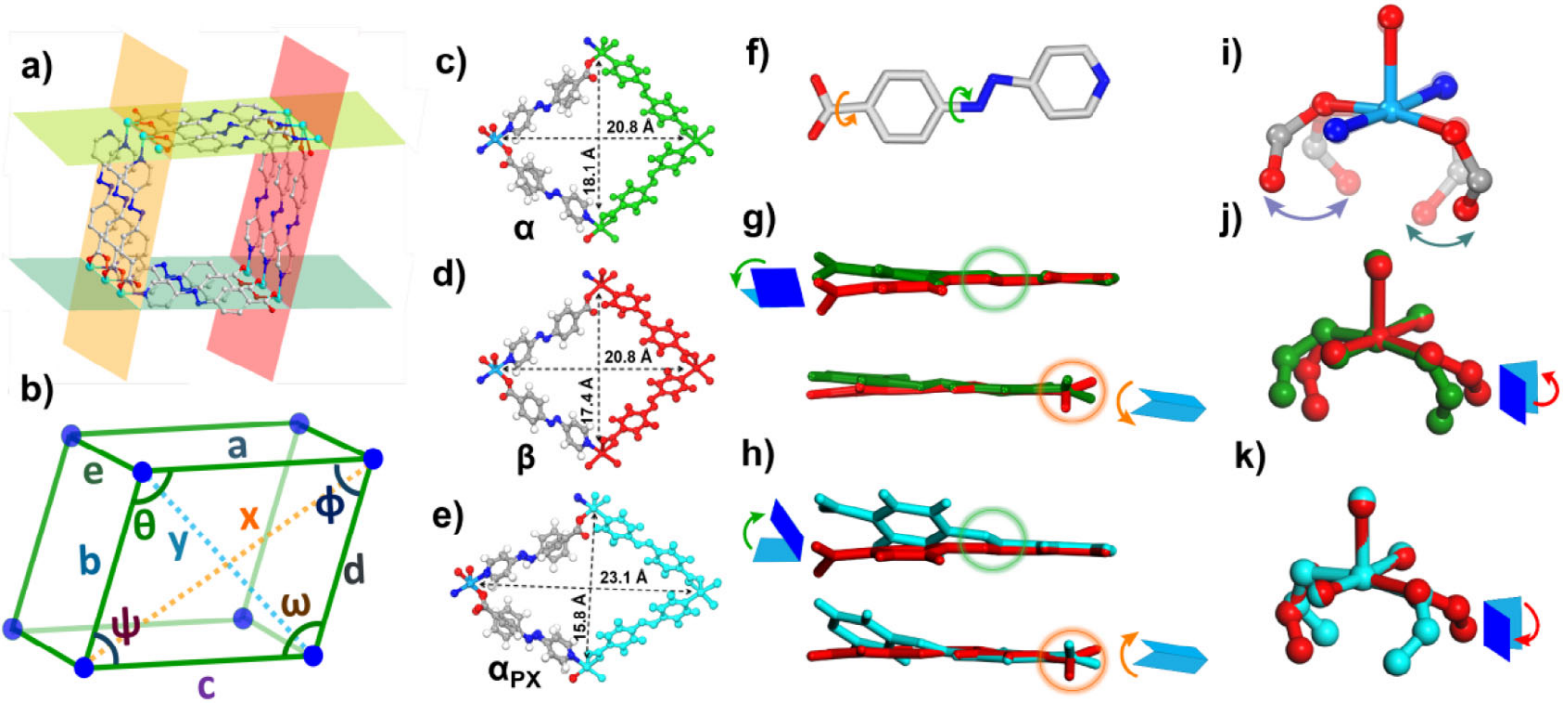
a) Dihedral angles between Cu cations at the vertices formed by planes of the pore walls and (b) distance and angle labels within each rhombic cavity of **X‐kdd‐1‐Cu**. Cross‐section of the rhombic channels and diagonal distances in (c) **X‐kdd‐1‐Cu‐α_DMF_** (green), (d) **X‐kdd‐1‐Cu‐β** and (red), and (e) **X‐kdd‐1‐Cu‐α_PX_** (blue) phase. (f) Deformation of ligand **1** around azo bond (green arrow) and a carboxyl group (orange arrow). (g) Comparison of superposed representations for deformations in ligand **1** in **X‐kdd‐1‐Cu‐α_DMF_** (green) versus **X‐kdd‐1‐Cu‐β** (red); (h) in **X‐kdd‐1‐Cu‐β** (red) versus **X‐kdd‐1‐Cu‐α_PX_** (blue). (i) Deformation of Cu‐based RBB. Comparison of superposed representations for deformations in Cu‐based RBB (j) in **X‐kdd‐1‐Cu‐α_DMF_** (green) versus **X‐kdd‐1‐Cu‐β** (red); (k) in **X‐kdd‐1‐Cu‐β** (red) versus **X‐kdd‐1‐Cu‐α_PX_** (blue).

PXRD studies indicated that, upon desolvation, the as‐synthesized phase **X‐kdd‐1‐Cu‐α_DMF_** underwent phase transformation to a smaller pore activated phase, **X‐kdd‐1‐Cu‐β**, while **X‐kdd‐2‐Cu** and **X‐kdd‐3‐Cu** did not transform (Figure S5). TGA analysis of activated **X‐kdd‐1‐Cu‐β** indicates full guest removal, with no significant mass loss observed below 260 °C (Figure S3). SCXRD studies revealed that **X‐kdd‐1‐Cu‐β** had indeed contracted with retention of space group and connectivity but slightly changed unit cell parameters and reduced cell volume (Table S2). Variable temperature PXRD (VT‐PXRD) tests revealed that the characteristic peak at 2θ  =  6.46° associated with **X‐kdd‐1‐Cu‐α_DMF_** at 298K had shifted to 2θ = 6.62° when the temperature was increased to 363K, i.e., after transformation to **X‐kdd‐1‐Cu‐β** (Figure S6). Additional phases observed above 363K were not further analyzed because of crystal fragmentation. For **X‐kdd‐2‐Cu** and **X‐kdd‐3‐Cu**, no significant peak shifts were observed (Figures S7, S8). SCXRD data revealed that the **α_DMF_** to **β** transformation in **X‐kdd‐1‐Cu** was accompanied by contraction of the channels with diagonal distance shortening and a decreased dihedral angle formed by the rhombic channel comprised of Cu centres in **X‐kdd‐1‐Cu‐α_DMF_** (Figure 3a–e, Tables S1, S4). Guest‐accessible volume decreased from 46.4% (**α_DMF_**) to 43.7% (**β**) (Table 1, Figure S9). The pore reduction from **α_DMF_** and **β** can be attributed to deformations in ligand **1** and the coordination environment of the Cu‐based RBBs (Figure 3 g,j).

**Table 1:** Diagonal distances between Cu cations, dihedral angles between Cu cations at the vertices formed by planes of the pore walls of the rhombic channel, cell volumes, and solvent‐accessible voids in five phases **X‐kdd‐1‐Cu‐α_DMF_, X‐kdd‐1‐Cu‐β, X‐kdd‐1‐Cu‐α_DCM_**, **X‐kdd‐1‐Cu‐α_PX_**, and **X‐kdd‐1‐Cu‐α_EB_**.

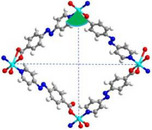	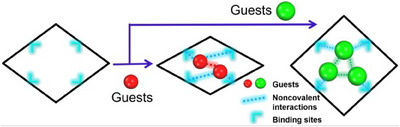			
	Diagonal distances (Å^2^)	Dihedral angles (average difference to 100°)	Cell volumes (Å^3^)	Voids (%)
**X‐kdd‐1‐Cu‐α_DMF_**	20.8 × 18.1	−2.1	3688.3(3)	46.4
**X‐kdd‐1‐Cu‐β**	20.8 × 17.4	−3	3427.4(5)	43.7
**X‐kdd‐1‐Cu‐α_DCM_**	22.9 × 15.7	11	3391.9(7)	43.4
**X‐kdd‐1‐Cu‐α_PX_**	23.1 × 15.8	11	3588.7(2)	46.4
**X‐kdd‐1‐Cu‐α_EB_**	21.2 × 18.2	−1.3	3765.7(2)	49.8

### Induced Fit Flexibility

To further explore the mechanism of flexibility in **X‐kdd‐1‐Cu**, crystals of **X‐kdd‐1‐Cu‐α_DMF_** were immersed in the following liquids (molecular volumes calculated by XSeed^[^ ^62^ ^]^): dichloromethane (DCM, 58 Å^3^); *para*‐xylene (PX, 133.2 Å^3^); ethylbenzene (EB, 130.6 Å^3^). SCXRD experiments revealed three new phases, **X‐kdd‐1‐Cu‐α_DCM_**, **X‐kdd‐1‐Cu‐α_PX_**, and **X‐kdd‐1‐Cu‐α_EB_**, which collectively indicate that **X‐kdd‐1‐Cu‐α** transforms its structure to accommodate each guest. As for **α_DMF_**, **α_DCM_**, **α_PX,_** and **α_EB_** also exhibited **kdd** topology, but the space group of **α_PX_** changed to *P1*, whereas the other phases retained space group *Cc*. The cell volumes are as follows: **α_DMF_**  =  3688.3(3) Å^3^; **β** = 3427.4(5) Å^3^; **α_DCM _** =  3391.9(7) Å^3^; **α_PX_** = 3588.7(2) Å^3^; **α_EB_** = 3765.7(2) Å^3^ (Tables 1 and S2). The corresponding solvent‐accessible void volumes were calculated to be 46.4%, 43.7%, 43.4%, 46.4%, and 49.8% for **α_DMF,_ β**, **α_DCM_**, **α_PX,_** and **α_EB_**, respectively (Table 1 and Figure S9). These results indicate that induced fit behavior occurs in **X‐kdd‐1‐Cu** thanks to its structural flexibility: DCM resulting in pore reduction from **β**; EB inducing pore expansion from the as‐synthesized phase. The diagonal distances and dihedral angles changed in the rhombic channels along *a*‐axis in all five phases (Table 1).

**Figure 4:**
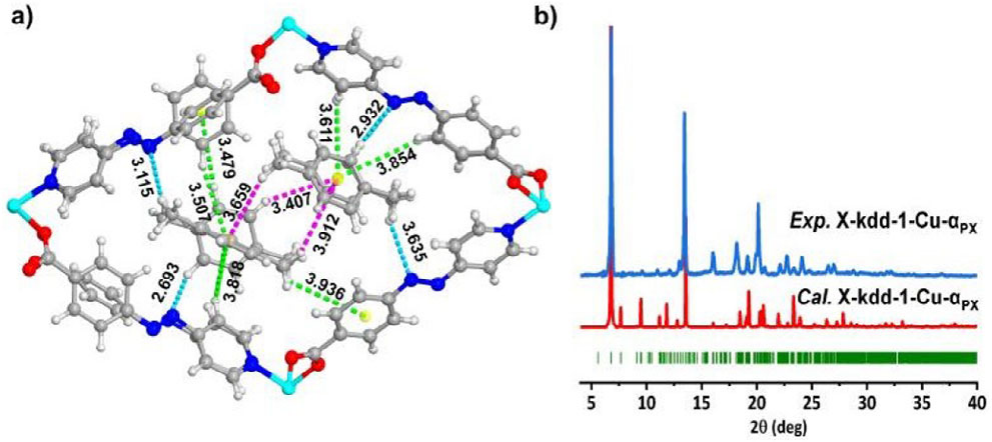
a) Structure of **X‐kdd‐1‐Cu‐α_PX_** with PX occupying site‐I with C−H⋅⋅⋅N hydrogen bonding interactions (blue dashed line) and site‐II with C‐H⋅⋅⋅ π interactions (green dashed line); C‐H⋅⋅⋅ π interactions between PX guests (magenta dashed line). The closest contacts between the framework and adsorbates are highlighted (Å). b) Comparison of experimental and calculated PXRD patterns from SCXRD‐determined structures for **X‐kdd‐1‐Cu‐α_PX_**.

Interestingly, even though the cell volumes and solvent‐accessible voids increased from **β** to **α_PX_** and **α_EB_**, the rhombic channel in **α_PX_** compresses compared to the **β** phase (diagonal distances in **α_PX _** = 23.1 × 15.8Å, **β** = 20.8×17.4Å) while expansion was observed from **β** to **α_EB_** (**α_EB_**: 21.2 × 18.2Å). FMOMs that undergo structural transformation to optimize binding site for a specific substrate are understudied and, to our knowledge, there are only nine previous reports of induced fit driven by guest sorption, with MIL‐53(Cr)^[^ ^63^ ^]^ being the first sorbent to transform from an activated large pore phase to a narrower pore phase (induced H_2_O, EtOH, MeOH).^[^ ^25^, ^26^, ^27^, ^28^, ^29^, ^30^, ^31^, ^32^, ^64^ ^]^ **X‐kdd‐1‐Cu** has a relatively large pore size (17.0Å with Cu atoms Vdw distances deducted) similar to that of the 13.3Å in MIL‐53(Cr). The guest‐host and guest‐guest interactions in **X‐kdd‐1‐Cu‐α_PX_**, as revealed by Figure 4a, there are two binding sites for PX. Site‐I involves multiple C─H⋅⋅⋅N hydrogen bonding interactions (D_N⋅⋅⋅H_ = 2.693, 2.932, 2.989, 3.115, 3.635Å), whereas site‐II exhibits multiple C─H⋅⋅⋅ π interactions (D_π⋅⋅⋅H_ = 3.478, 3.507, 3.517, 3.611, 3.818, 3.854, 3.936Å) with the pore walls (Figure 4a). C─H⋅⋅⋅ π interactions were also observed between PX guests (D_π⋅⋅⋅H_ = 2.679, 3.305, 3.407, 3.659, 3.912Å). These different host–guest/guest–guest interactions are key to driving the transformation of **X‐kdd‐1‐Cu‐β** to **X‐kdd‐1‐Cu‐α_PX_**. In effect, the azo bond in ligand **1** can act as an axle to enable flexibility (Figure 3f), and indeed deformations were observed in **1** around the azo bond/carboxyl groups. This, in turn, allowed adjacent ligands in **1** to change from parallel (face‐to‐face) to angled (edge‐to‐face) stacking interactions. The dihedral angle of planes formed by the benzoate ring and pyridine ring in **β** and **α_PX_** thereby changed from 4.98∼5.76° to 1.38°∼31.22°, a maximum difference of ∼26° (Figure 3h, Table S4). The C‐C bond connecting the carboxyl group and phenyl ring was also found to be twisted in **β** versus **α_PX_**, the maximum dihedral angle difference being ∼24° (Figure 3g and Table S4). The Cu^2+^ cations in **α_PX_** were found to be different than the other four phases, with alternating octahedral and tetrahedral geometries, thanks to the torsion angle of the carboxyl group (Figures 3i,k and S10). When the Cu and N atoms of the Cu node are lefted and superimposed, the maximum ∠C**α_PX_**‐Cu‐C**β** measured between **β** and **α_PX_** is ∼31° (Figure 3k). Overall, deformations of both the Cu‐based RBB and ligand **1** together enabled compression of the channel in **α_PX_**. **X‐kdd‐1‐Cu‐α_DCM_** was also found to exhibit induced fit with channel shrinkage from **β** via loading of DCM guests (diagonal distances changed from 20.8×17.4 Å^2^ in **β** to 22.9×15.7 Å^2^, Table 1), resulting in reduced cell volume and solvent‐accessible void volume in **α_DCM_** versus **β**. Ligand **1** and the Cu coordination environment both underwent deformation (Figure S11).

FT‐IR studies of **α_PX_** and **α_EB_** are consistent with loading of C8 guests (aromatic ring stretching bands at 3046 cm^−1^ to 2861 cm^−1^) and loading of DCM in **α_DCM_** was supported by C─H stretching bands at 3049 cm^−1^ (Figure S4). SCXRD data revealed that both the RBB and azo linker in these three phases had undergone conformational changes compared to **X‐kdd‐1‐Cu‐α_DMF_** and **β**. PXRD experiments conducted upon the PX, EB, and DCM loaded phases are consistent with bulk purity (Figures 4b and S12). PX, MX, and OX sorption isotherms were measured at 298K (P/P_0_ = 0–95%) on **X‐kdd‐1‐Cu**, revealing similar Type I profiles with strong hysteresis. These isotherms do not indicate selective sorption of one of the isomers (Figure S13). Conversely, exposure under the same conditions did not induce significant phase changes in **X‐kdd‐2‐Cu** or **X‐kdd‐3‐Cu** (Figures S14, S15). The reversible nature of the structural flexibility of **X‐kdd‐1‐Cu** is indicated by PXRD data collected after three consecutive cycling experiments that involved alternating DCM soaking and activation (Figure S16).All three variants were found to be unstable to soaking in water (Figure S16).

### Gas Sorption and In Situ PXRD Analysis

The differences in flexibility between **X‐kdd‐1‐Cu**, **X‐kdd‐2‐Cu,** and **X‐kdd‐3‐Cu** motivated us to conduct CO_2_ and C2 gas sorption to probe if they can also induce structural transformations.^[^ ^65^ ^]^ CO_2_ sorption conducted at 195K (Figure S17a) revealed different isotherm profiles with **X‐kdd‐1‐Cu** exhibiting a stepped isotherm. In addition to small uptake in the low‐pressure region (P/P_0_ = 0–0.1) of the isotherms of all three compounds, **X‐kdd‐1‐Cu** displayed an additional step at P_GO_ = 0.32 (Figure S17a) consistent with a structural transformation. For **X‐kdd‐1‐Cu**, **X‐kdd‐2‐Cu,** and **X‐kdd‐3‐Cu**, Langmuir surface areas calculated from the CO_2_ sorption isotherms at 195K were determined to be 1729, 1838, and 2779 cm^2^g^−1^, respectively. Pore volumes of **X‐kdd‐1‐Cu**, **X‐kdd‐2‐Cu**, and **X‐kdd‐3‐Cu** calculated from their 195K CO_2_ sorption isotherm by assuming liquid filling of CO_2_ when saturated at P/P_0_ = 0.95 were 0.50, 0.55, and 0.60 cm^3^g^−1^, respectively. These values are consistent with the pore volumes estimated from their crystal structures: 0.49, 0.62 (simulated structure), and 0.57 cm^3^g^−1^, respectively. For the desorption isotherms, only **X‐kdd‐1‐Cu** exhibited hysteresis with a gate closing pressure around P/P_0_ = 0.37, while the desorption isotherms of **X‐kdd‐2‐Cu** and **X‐kdd‐3‐Cu** coincided with their adsorption isotherms. Three cycles of CO_2_ activation/sorption were conducted at 195K on the same sample of **X‐kdd‐1‐Cu** and revealed consistent isotherm profiles, further indicating that **X‐kdd‐1‐Cu** is recyclable (Figure S18). The N_2_ uptake of **X‐kdd‐1‐Cu** and **X‐kdd‐2‐Cu** were found to be 21 and 81 cm^3^/g, respectively (Figure S19). Significant N_2_ uptake was not observed in **X‐kdd‐3‐Cu** as reported previously.^[^ ^58^, ^59^ ^]^ As noted by a reviewer, the CO_2_ adsorption capacities at 195K of **X‐kdd‐1‐Cu**, **X‐kdd‐2‐Cu**, and **X‐kdd‐3‐Cu** are consistent with structural, in situ PXRD and VT‐PXRD experiments, whereas the N_2_ uptakes at 77K are anomalously low. Whereas one can generally expect differences between N_2_ and CO_2_ sorption profiles, especially for flexible and ultramicroporous sorbents, because of their different physicochemical properties,^[^ ^65^ ^]^ this data cannot be readily explained for larger pore structures that show no evidence of guest‐induced flexibility. Nevertheless, although N_2_ uptake at 77K is anomalous for all three compounds, data for other gases studied are consistent with the SCXRD and PXRD data.

C2 gas sorption isotherms also suggested flexibility of **X‐kdd‐1‐Cu**. Whereas sorption conducted at 298K showed nearly linear Type I isotherms for **X‐kdd‐1‐Cu**, **X‐kdd‐2‐Cu** and **X‐kdd‐3‐Cu** (Figure S20), at 273 K **X‐kdd‐1‐Cu** exhibited stepped isotherms with the following gate opening pressures: C_2_H_2_, P_GO_ = 347 mmHg; C_2_H_4_, P_GO_ = 686 mmHg; C_2_H_6_, P_GO_ = 477 mmHg (Figure S17b–d). **X‐kdd‐2‐Cu** and **X‐kdd‐3‐Cu** retained their almost linear Type I isotherms.

**Figure 5:**
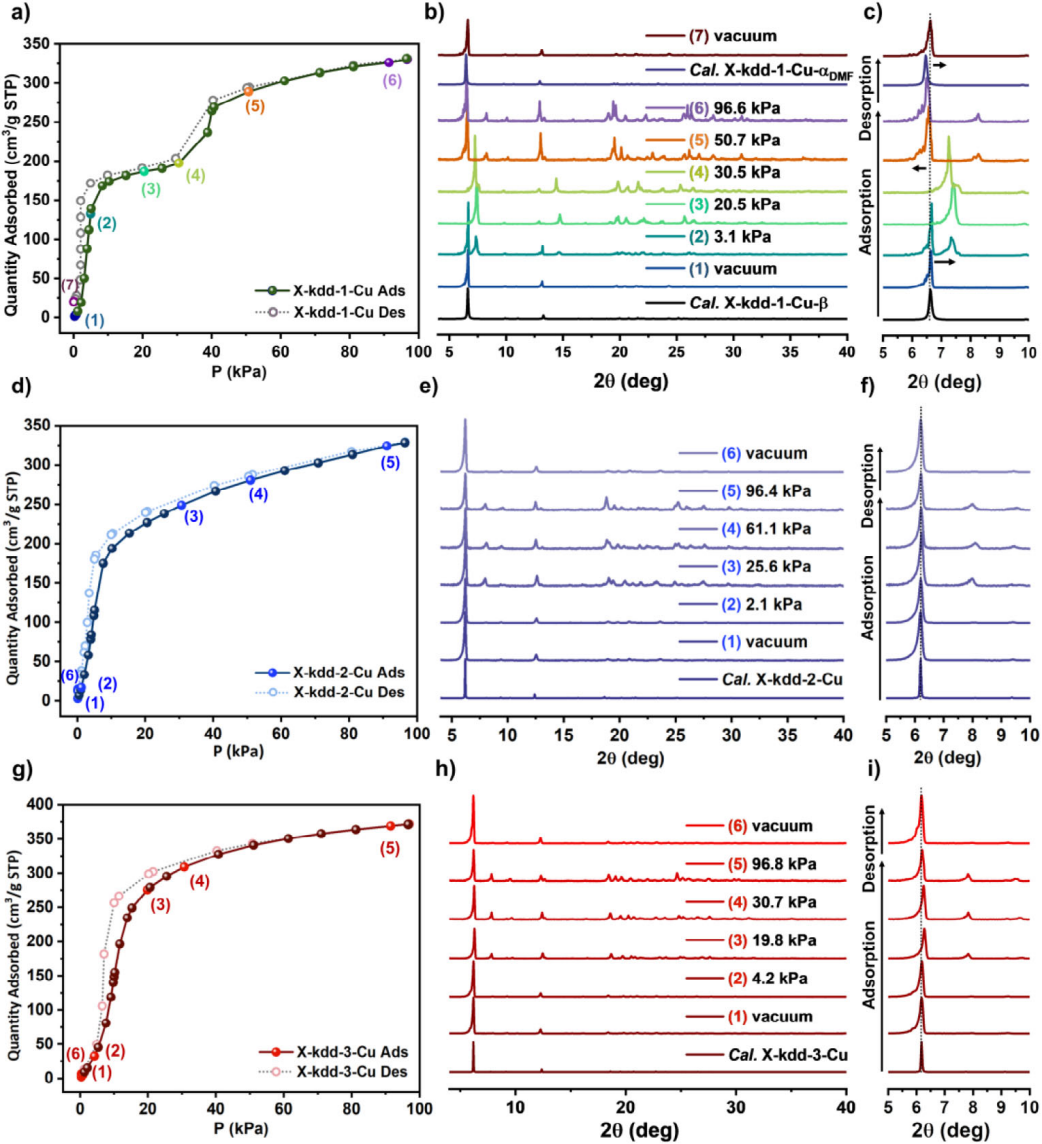
CO_2_ sorption isotherms for a) **X‐kdd‐1‐Cu**, (d) **X‐kdd‐2‐Cu** and (g) **X‐kdd‐3‐Cu** at 195 K. Corresponding in situ CO_2_‐loaded PXRD patterns for (b) **X‐kdd‐1‐Cu** (e) **X‐kdd‐2‐Cu,** and (h) **X‐kdd‐3‐Cu** at various adsorption (ads; closed sphere) and desorption (des; open sphere) points. Magnified PXRD patterns from 5 to 10° 2θ values for (c) **X‐kdd‐1‐Cu**, (f) **X‐kdd‐2‐Cu,** and (i) **X‐kdd‐3‐Cu**.

In situ variable‐pressure PXRD and sorption coincident measurements conducted with CO_2_ from 0 to 1 bar at 195K revealed that **X‐kdd‐1‐Cu** indeed underwent reversible structural changes, while the other two sorbents did not exhibit phase transformations (Figure 5a–i). In the CO_2_ adsorption process of **X‐kdd‐1‐Cu**, the distinct low angle peak of the starting material **X‐kdd‐1‐Cu‐β** (6.62)° diminished as the peak of **X‐kdd‐1‐Cu‐α_DMF_** (6.46)° appeared at saturated CO_2_ (96.6 kPa, 0.95 bar) pressure (Figure 5b,c), indicative of phase transformation from **β** to **α_DMF_**. As for the VT‐PXRD data, intermediate phases were observed between 4.8–50.7 kPa (0.05–0.5 bar) (Figure 5b,c). The in situ PXRD patterns of the desorption process indicate that the CO_2_ loaded phase **α_DMF_** reverted to **β**, suggesting reversibility. In contrast, for **X‐kdd‐2‐Cu**, the characteristic PXRD peaks did not shift during CO_2_ sorption (Figure 5e,f), while **X‐kdd‐3‐Cu** exhibited only minor peak shifts (Figure 5 h,i). The PXRD patterns of both **X‐kdd‐2‐Cu** and **X‐kdd‐3‐Cu** indicate structural rigidity relative to **X‐kdd‐1‐Cu**. Nevertheless, SCXRD analysis of **X‐kdd‐3‐Cu** as synthesized and after PX‐soaking revealed slight structural deformation of the linker ligand that did not result in a significant change in structural parameters (Table S5). We attribute this lack of flexibility to the linear geometry of ligand **3**. This contrasts with the pronounced structural changes observed for **X‐kdd‐1‐Cu** (Table S6).

### Computational Studies

**Figure 6:**
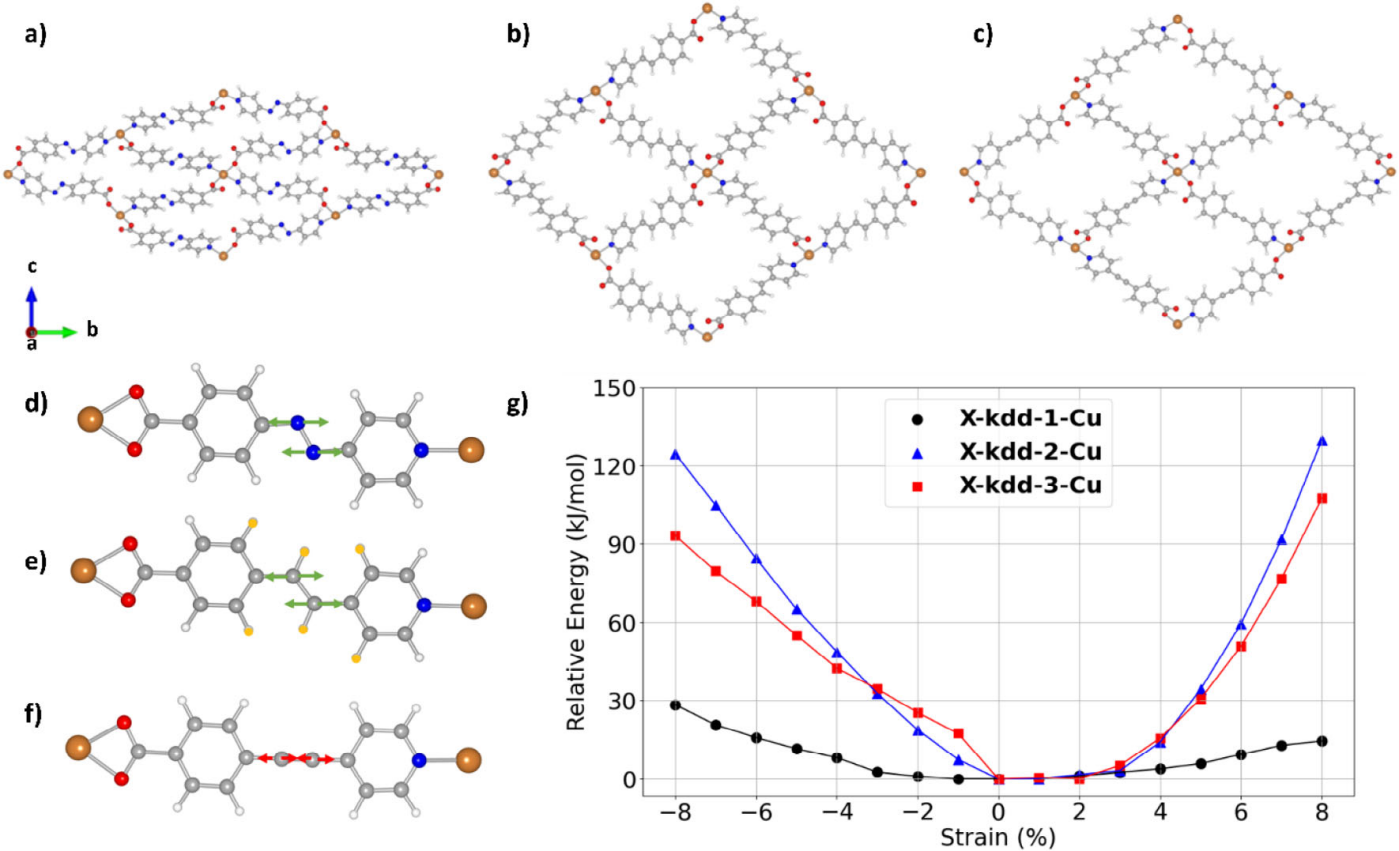
DFT relaxed structures: a) **X‐kdd‐1‐Cu**, b) **X‐kdd‐2‐Cu**, and c) **X‐kdd‐3‐Cu**. Potential deformations during strain analysis which illustrated by linkers **1**–**3** in: d) **X‐kdd‐1‐Cu**, e) **X‐kdd‐2‐Cu,** and f) **X‐kdd‐3‐Cu** (for clarity, only the linker is shown, but the fully periodic structure was modeled). g) Potential energy versus strain for all three compounds. (Color code: orange = Cu, red = O, grey = C, white = H, and blue = N.)

To gain further insight into the mechanism of flexibility in **X‐kdd‐n‐Cu** (**n** = **1**, **2**, **3**), ab initio calculations (see Supporting Information) were conducted. The energy of linker rotation/bending and whole framework deformation was modeled. The only difference in the three CNs is the central bond in L. Linker rotation along the central axis of benzoic rings (Figure S21) and bending along the vertical axis through the central bonds (Figure S22) in linkers **1**–**3** from −60 to + 60 degrees are displayed. Little variance of relative energy in this mode of flexibility between the three linkers was seen. However, ab initio relaxations reveal that the whole structure of **X‐kdd‐1‐Cu** offers closed pores when compared to the relaxed structures of **X‐kdd‐2‐Cu** and **X‐kdd‐3‐Cu** (Figure S6a–c). A strain analysis was performed by stretching and compressing the relaxed structures along the *c*‐axis. Each structure was stretched and compressed up to 8% in steps of 1%, and the cell volume was held constant while the atomic positions within the structures were allowed to relax using the minimization algorithms within VASP.^[^ ^66^, ^67^ ^]^ Linkers **1**–**3** in the three relaxed structures can explain the variations in flexibility (Figures 6d–f and S23). Stretching and compressing of **X‐kdd‐3‐Cu** is unlikely because it necessitates shortening or elongation of the carbon triple bond, which is energetically expensive. For **X‐kdd‐1‐Cu** and **X‐kdd‐2‐Cu,** these deformations do not necessitate a change in any bond lengths, but in **X‐kdd‐2‐Cu,** the hydrogen atoms highlighted in yellow will begin to interact unfavorably once enough compression or stretching occurs. This renders stretching and compression energetically unfavorable in **X‐kdd‐2‐Cu**. **X‐kdd‐1‐Cu** is the only linker that enables this form of deformation with a low energy penalty (Figure 6g). These results are consistent with the experimental crystallographic data.^[^ ^68^ ^]^ Since the ground‐state energy of **X‐kdd‐1‐Cu** is comparable to the energies of deformations about those minima, the ability of **X‐kdd‐1‐Cu** to form multiple additional phases of **X‐kdd‐1‐Cu** can be explained. Conversely, **X‐kdd‐2‐Cu** and **X‐kdd‐3‐Cu** are much more resistant to deformations. The elastic moduli tensors for **X‐kdd‐1‐Cu** and **X‐kdd‐3‐Cu** were calculated to further assess the flexibility of these CNs. A lower bulk modulus in **X‐kdd‐1‐Cu** than **X‐kdd‐3‐Cu** was obtained (Table S7), further supporting the flexibility of **X‐kdd‐1‐Cu**.

## Conclusion

We report that an isostructural family of three Cu‐based RBB CNs exhibits different propensities for structural flexibility. While **X‐kdd‐1‐Cu** exhibited induced fit binding towards multiple guests, **X‐kdd‐2‐Cu** and **X‐kdd‐3‐Cu** retained their as‐synthesized phases. We attribute the propensity for induced fit behavior by **X‐kdd‐1‐Cu** to the diazo moiety in its linker ligand. According to computational studies, ligand **1** exhibits a lower deformation energy barrier, which in turn enables a lower bulk modulus for **X‐kdd‐1‐Cu**, results in a rare example of induced fit whereby DCM guests induce contraction of the already contracted activated phase. This contribution, therefore, not only affords insight into mechanisms of induced fit behavior in CNs but also provides a general crystal engineering principle to obtain flexible CNs.

## Conflict of Interests

The authors declare no conflict of interest.

## Supporting information



Supporting Information

Supporting Information

## Data Availability

The data that support the findings of this study are available in the Supporting Information of this article.
