# Supplementary material for: A Diazo Linker Ligand Promotes Flexibility and Induced Fit Binding in a Microporous Copper Coordination Network
Source: Angew Chem Int Ed Engl. 2025 Jun 10;64(31):e202507757. doi: 10.1002/anie.202507757 (PMC12304801; doi:10.1002/anie.202507757)
Supplement: Supplementary file 1 — Supporting Information [file ANIE-64-e202507757-s002.pdf]

Supporting Information

A Diazo Linker Ligand Promotes Flexibility and Induced Fit Binding in a Microporous Copper Coordination Network

Xia Li,[a,b] Debobroto Sensharma,[b] Wells Graham,[c] Volodymyr Bon,[d] En Lin,[a] Xiang-Jing Kong,[b] Tao He,[b] Andrey A. Bezrukov,[b] Zhenjie Zhang,[a] Stefan Kaskel,[d] Timo Thonhauser,[c] Michael J. Zaworotko[b]*

[a]College of Chemistry, Nankai University, Tianjin 300071, People's Republic of China
[b]Department of Chemical Science, Bernal Institute, University of Limerick, Limerick, V94 T9PX, Republic of Ireland
[c]Department of Physics and Center for Functional Materials, Wake Forest University, Winston-Salem, North Carolina 27109, United States
[d]Faculty of Chemistry, Technische Universität Dresden, Bergstrasse 66, 01062 Dresden, Germany

Context

1. Materials and Synthesis S1
2. Single-crystal X-ray diffraction measurements. S2
3. Thermogravimetric analysis (TGA) S2
4. IR spectra Fourier Transform Infrared (FTIR) Spectroscopy S3
5. Powder X-ray diffraction measurements S3
6. Variable Temperature Powder X-ray Diffraction (VT-PXRD) S3
7. *In suit* CO_2 loaded Powder X-ray Diffraction. S3
8. Gas sorption measurements. S4
9. Computational simulation. S4
10. Supporting Figures and Tables S6
11. References S39

1. Materials and Synthesis

4-aminopyridine, 4-aminobenzoic acid and $Cu(NO_3)_2{\cdot}3H_2O$ were obtained commercially and used as received without further purification. Synthesis of ligand **1** was accomplished by a modification of a previously reported procedure.[1] Synthesis of ligands **2**, and **3** was accomplished by applying previously reported procedures.[2,3]

(*E*)-4-(pyridin-4-yldiazenyl) benzoic acid (1): To a solution of 4-aminobenzoic acid (1.0 g, 7.3 mmol) in dichloromethane (12mL), an aqueous solution of Oxone® (8.97 g in 45 mL deionized water, 14.6 mmol), was added and the suspension vigorously stirred at room temperature for 1 hour. 4-nitrosobenzoic acid precipitated as a pale solid was isolated by filtration, washed with water, and dried in an 80 °C oven (1.1 g, yield = quant.). Sodium hydroxide (3.00 g, 75 mmol) was dissolved in 100 mL of deionized water to form a 3% NaOH solution. 4-aminopyridine (0.53g, 5.6 mmol) and 4-nitrosobenzoic acid (0.45g, 3.0 mmol) were added and the mixture was heated to reflux overnight. The resulting bright orange solution was then cooled to room temperature, forming a bright orange needle precipitate. Then dissolve the orange precipitate in 30mL water and adjust the pH = 1 with 1M HCl. The resulting precipitate was filtered, washed with water, and dried in a vacuum to give the product (0.5g, 2.2mmol, 73%) a light orange solid. ^{1}H NMR (400 MHz, DMSO) δ 13.37 (s, 1H), 8.88 (d, *J* = 6.1 Hz, 2H), 8.19 (d, *J* = 8.7 Hz, 2H), 8.05 (d, *J* = 8.7 Hz, 2H), 7.81 (d, *J* = 6.1 Hz, 2H).

X-kdd-1-Cu-α_{DMF}, [Cu(1)$_2$]$_n$-α_{DMF}:
Synthesis: A mixture of $Cu(NO_3)_2{\cdot}3H_2O$ (0.05 mmol, 12.1 mg), **1** (0.1 mmol, 22.7 mg), *N,N*-Dimethylformamide (DMF, 6 mL) and ethanol (EtOH 1mL) were added to a 20 mL glass vial. The vial was capped tightly and placed in an oven at 85 °C for 24h, which was then cooled to room temperature. After rinsing several times with fresh DMF, brown single crystals were obtained. Yield, 80% based on copper.

X-kdd-1-Cu-β, [Cu(1)$_2$]$_n$-β:
Synthesis: The as-synthesized framework (**X-kdd-1-Cu-α_{DMF}**) was exchanged with fresh dichloromethane (DCM) by using a Soxhlet extractor for 2 days and then heated to 60 °C under vacuum for 2h to yield **X-kdd-Cu-1-β**.

X-kdd-1-Cu-α_{DCM}, [Cu(1)$_2$]$_n$-α_{DCM}:
Synthesis: The as-synthesized open framework (**X-kdd-1-Cu-α_{DMF}**) was exchanged with fresh DCM for 3 days to yield **X-kdd-Cu-1-α_{DCM}**.

X-kdd-1-Cu-α_{PX}, [Cu(1)$_2$]$_n$-α_{PX}:
Synthesis: The as-synthesized open framework (**X-kdd-1-Cu-α_{DMF}**) was exchanged with fresh *para*-xylene (PX) for 3 days to yield **X-kdd-Cu-1-α_{PX}**.

X-kdd-1-Cu-α_{EB}, [Cu(1)$_2$]$_n$-α_{EB}:
Synthesis: The as-synthesized open framework (**X-kdd-1-Cu-α_{DMF}**) was exchanged with fresh ethylbenzene (EB) for 3 days to yield **X-kdd-1-Cu-α_{EB}**.

X-kdd-2-Cu, $[Cu(2)_2]_n$:

Synthesis: A mixture of $Cu(NO_3)$ $_23H_2O$ (0.05 mmol, 12.1 mg), **2** (0.1 mmol, 22.4 mg), N,N-Dimethylformamide (DMF, 6 mL) and ethanol (EtOH 2 mL) were added to a 20 mL glass vial. The vial was capped tightly and placed in an oven at 85 °C for 24h, which was then cooled to room temperature. After rinsing several times with fresh DMF, the light blue powder was obtained. Yield, 78% based on copper.

X-kdd-3-Cu ($Cu(pbea)_2$),[3,4] **$[Cu(3)_2]_n$:**

Synthesis: The synthesis procedure followed the reported method.[3,4] $Cu(NO_3)_2\cdot 3H_2O$ (6.04 mg, 0.025 mmol) and **3** (11 mg, 0.05 mmol) were mixed with 2 mL DMF and 1 mL ethanol. The mixture was sealed in a vial and heated to 90 °C for 48 h, followed by cooling to ambient temperature. After filtering, the product was exchanged with DCM for 4 days. Activated **X-kdd-3-Cu** was obtained after the solvent-exchanged sample was evacuated at 40°C for 20 hours.

2. Single-crystal X-ray diffraction measurements.

Single-crystal reflection data were collected on a Bruker Quest diffractometer equipped with a CMOS detector and IμS microfocus X-ray source (Cu K_α, λ = 1.54178 Å; Mo K_α, λ = 0.71073 Å). Indexing was performed using APEX3[5] (Difference Vectors method). Absorption correction was performed by the multi-scan method implemented in SADABS.[6] Space group was determined using XPREP implemented in APEX3.[5] Structural solution and refinement against F^2 were carried out using the SHELXL non-linear least squares implemented in Olex2 v1.2.10.[7,8] All non-hydrogen framework atoms were refined with anisotropic parameters, while H atoms were placed in calculated positions and refined using a riding model. Some disordered atoms have been refined isotropically. All the crystals were measured under liquid N_2 flow at a temperature of 100K or 110K to avoid the phase transformation caused by guest molecules escaping in the air. To obtain single-crystal X-ray diffraction (SCXRD) data of **X-kdd-Cu-1-β**, as-synthesized **X-kdd-Cu-1** was solvent-exchanged with DCM and activated at 60 °C under vacuum for 2h using a 3Flex vacuum station with a long-neck tube. After activation, the tube was purged with N_2 to prevent air/moisture exposure, and the sample was coated with oil prior to mounting on the goniometer. Crystallographic data and structural refinement information are listed in Tables S1. The structure of phases **X-kdd-1-Cu-α_{DMF}, β, α_{DCM},** and **α_{EB}** was solved and refined in the *Cc* space group; **X-kdd-1-Cu-α_{PX}** in the *P1* space group. Crystallographic data for the structures reported in this paper have been deposited with the Cambridge Crystallographic Data Centre as supplementary publication Nos. CCDC 2244644 (for **X-kdd-1-Cu-α_{EB}**), 2244645 (for **X-kdd-1-Cu-α_{DCM}**), 2244646 (for **X-kdd-1-Cu-β**), 2244647 (for **X-kdd-1-Cu-α_{PX}**), 2244648 (for **X-kdd-1-Cu-α_{DMF}**), 2447686 (for **X-kdd-3-Cu (PX)**), 2447687 (for **X-kdd-3-Cu (as-synthesized)**).

3. Thermogravimetric analysis (TGA)

Thermogravimetric analyses (TGA) were performed under N_2 using a TA Instruments Q50 system. Samples were loaded into aluminum sample pans and heated at 10 K min^{-1} from room temperature to 500 °C.

4. IR spectra Fourier Transform Infrared (FTIR) Spectroscopy

Spectra were obtained by using a FTIR spectrometer (Agilent technologies, Cary 630) in the range of 4000-650 cm^{-1}.

5. Powder X-ray diffraction measurements

Powder X-ray diffraction patterns were recorded on a PANalytical Empyrean™ diffractometer equipped with a PIXcel3D detector, operating in scanning line detector mode with an active length of 4 utilizing 255 channels, in the Continuous Scanning mode with the goniometer in the theta-theta orientation. The diffractometer is fitted with an Empyrean Cu LFF (long fine-focus) HR (9430 033 7310x) tube operated at 40 kV and 40 mA, and CuKα radiation (λα = 1.540598 Å) was used for diffraction experiments. Incident beam optics included the Fixed Divergences slit with anti-scatter slit PreFIX module, with a 1/8° divergence slit and a 1/4° anti-scatter slit, as well as a 10 mm fixed incident beam mask and a Soller slit (0.04 rad). Divergent beam optics included a P7.5 anti-scatter slit, a Soller slit (0.04 rad), and a Ni-β filter. The data were collected in the range of 2θ = 3 – 40°. Raw data were then evaluated using the X'Pert HighScore Plus™ software V 4.1 (PANalytical, The Netherlands).

6. Variable Temperature Powder X-ray Diffraction (VT-PXRD)

Diffractograms at different temperatures were recorded using a PANalytical X'Pert Pro-MPD diffractometer equipped with a PIXcel3D detector operating in scanning line detector mode with an active length of 4 utilizing 255 channels. The Anton Paar TTK 450 stage and the Anton Paar TCU 110 Temperature Control Unit were used to record the variable temperature diffractograms. The diffractometer is outfitted with an Empyrean Cu LFF (long fine focus) HR (9430 033 7300x) tube operated at 40 kV and 40 mA and CuKα radiation (λ_α = 1.54056 Å) was used for diffraction experiments. The data was collected by continuous scanning mode with the goniometer in the theta-theta orientation. Incident beam optics included the Fixed Divergences slit, with a 1/4° divergence slit and a Soller slit (0.04 rad). Divergent beam optics included a P7.5 anti-scatter slit, a Soller slit (0.04 rad), and a Ni-β filter. In a typical experiment, 20 mg of sample was ground into a fine powder and loaded on a zero-background sample holder made for Anton Paar TTK 450 chamber. The data were collected from 4 - 40° (2θ) with a step size of 0.0167113° and a scan time of 50 seconds per step. Crude data were analyzed using the X'Pert HighScore Plus™ software V 4.1 (PANalytical, The Netherlands). The sample was heated up to 523 K.

7. *In suit* CO_2 loaded Powder X-ray Diffraction.

In situ PXRD patterns on **X-kdd-n-Cu** (**n** = **1**, **2**, **3**) in parallel to CO_2 physisorption at 195K, respectively, were measured using home-built dedicated instrumentation, based on Empyrean-2 powder X-ray diffractometer (ω–2θ goniometer, alpha1 system) using a customized setup based

on ARS DE-102 closed cycle helium cryostat (T = 30–300 ± 0.1 K) and adsorption cell, built of 1.33'' CF-flange and Beryllium dome. The cell was connected to the low-pressure port of the BELSORP-max (Microtrac MRB) volumetric adsorption instrument. The TTL-trigger was used for establishing the communication between BELSORP-max and Data Collector software and ensure the measurement of adsorption isotherm and PXRD patterns in automated mode. The diffraction experiments were performed using ω–2θ scans in transmission geometry in the range of 2θ = 3–40°. Parallel beam optics (W/Si mirror, hybrid 2xGe(220) monochromator, 4 mm mask, primary divergence and secondary antiscatter slits with ¼° opening) was used for the data collection. Pixcel-3D detector in 1D scanning mode (255 active channels) was used for recording of the scattered intensities. A physisorption of CO_2 at temperature 195K and 111K, respectively, was measured on 60 mg of **X-kdd-n-Cu** (**n** = **1**, **2**, **3**) sample, mounted in the X-ray beam, and PXRD patterns were recorded after equilibration (0.1% of pressure change within 300 s) at selected points of the isotherm. Adsorption and desorption isotherm, measured *in situ* and corresponding PXRD patterns are given in the Figure 5.

8. Gas sorption measurements.

For gas sorption experiments, high-purity gases were used as received from BOC Gases Ireland: CO_2 (99.995%), N_2 (99.9995%), C_2H_2 (98.5%), C_2H_4 (99.92%), C_2H_6 (99.0%). A Micromeritics 3Flex surface area and pore size analyzer 3500 was used for collecting the low-pressure sorption isotherms for CO_2 and N_2. The temperature at 77 K was maintained using a 4 L Dewar filled with liquid nitrogen. The temperature at 195 K was maintained using a 4 L Dewar filled with a dry ice-acetone mixture. Bath temperatures of 273 and 298 K were precisely controlled with a Julabo ME (v.2) recirculating control system containing a mixture of ethylene glycol and water. dichloromethane (DCM) exchanged **X-kdd-n-Cu** (**n** = **1**, **2**, **3**) was degassed under a high vacuum at 60 °C for 10 h on a Micromeritics Smart VacPrep instrument. The activated sample (100 mg) was transferred to 3Flex and evacuated at room temperature for 2 hours before the measurements.

9. Computational simulation.

Pawley fits of PXRD of ***X-kdd-2-Cu***

Unit cell determinations for **X-kdd-2-Cu** were carried out using the X-Cell program embedded in Materials Studio. The low-angle tail of the dominating reflection is distorted by the influence of axial divergence. Lattice parameters and space group were determined from a set of 17 reflections. A monoclinic unit cell with a space group of *Cc* (No.9) and lattice constants of a = 5.10 Å, b = 43.54 Å, c = 18.87 Å, α = γ = 90°, β = 95.65°was able to index. Given the structure and lattice parameters of **X-kdd-1-Cu**, an isoreticular structure model was constructed and further optimized with density functional theory (DFT) calculations within fixed lattice constants using the DMol3 program in the Materials Studio. The GGA-PBE functional and DNP basis sets were used for our calculations. Finally, the Pawley refinement was performed, which resulted in cell unit parameters a = 5.12 Å, b = 43.61 Å, c = 18.96 Å, α = γ = 90°, β = 95.77°(R_p = 4.30%, R_{wp} = 8.51%). The refinement results confirmed that the framework structure of **X-kdd-2-Cu** was isoreticular with that of **X-kdd-1-Cu**.

Energy calculation of frameworks and ligands

To understand the different degrees of flexibility within the three structures, we conducted *ab*

initio calculations using VASP.[9,10] The VASP default PAW pseudopotentials were utilized along with the vdW-DF functional.[11–14] A k-point grid of $2 \times 2 \times 1$ was sampled for **X-kdd-1-Cu**, while a grid of $2 \times 1 \times 1$ was used for **X-kdd-2-Cu** and **X-kdd-3-Cu**. A kinetic energy cutoff of 600 eV was used to ensure well-converged results. SCF loops were carried out until the accuracy was within 10^{-5} eV. Geometric optimization was performed until the forces on all atoms were at or below 0.005 eV/Å. The Hubbard U value assigned for copper was 4.[15] Once the structures were fully relaxed, energy-strain curves were calculated to uncover the mechanism behind the pore opening phenomenon that was experimentally observed. The elastic moduli tensor was calculated as well using the finite difference method implemented in VASP. The rotation and bending deformation response of the linkers were also studied (as outlined in Figures S21-S23 and Table S7).

10. Supporting Figures and Tables

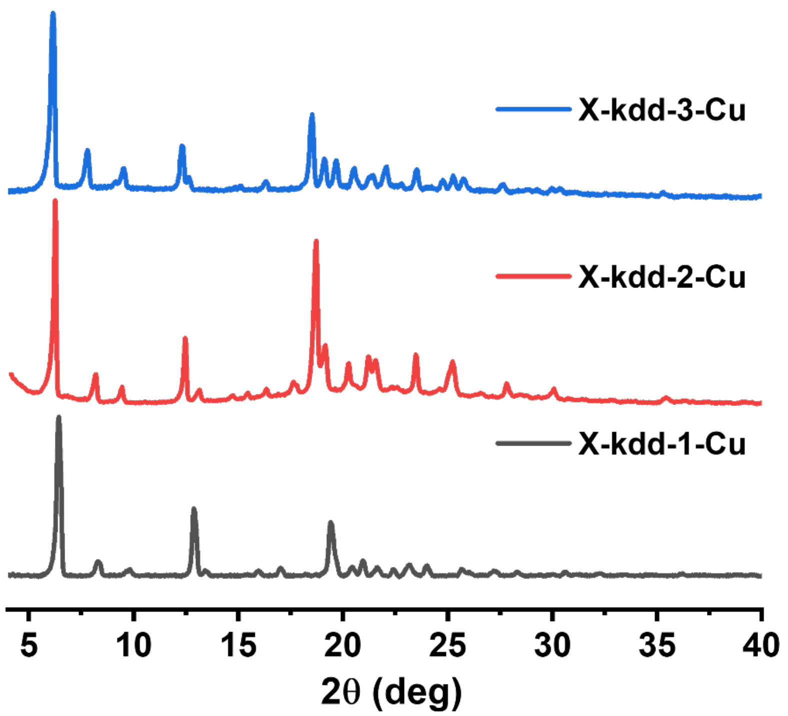

Figure S1. PXRD patterns of as-synthesized **X-kkd-1-Cu**, **X-kkd-2-Cu**, **X-kkd-3-Cu**.

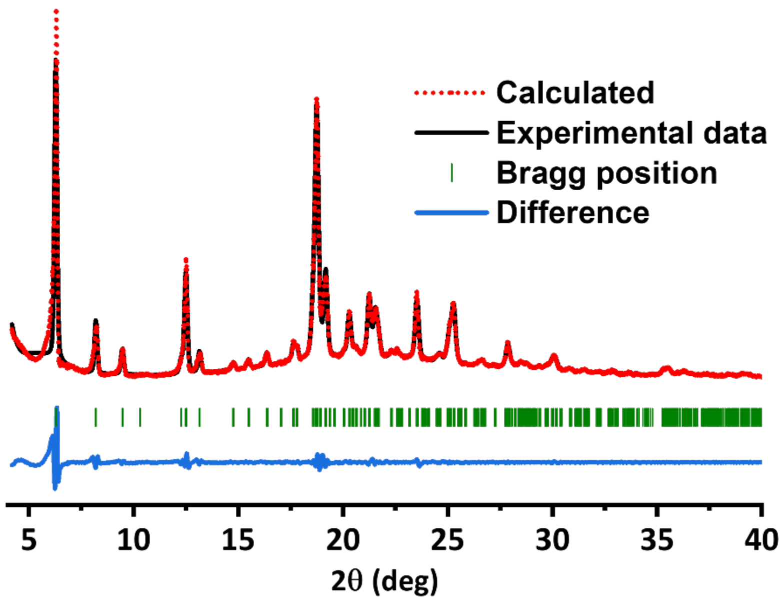

Figure S2. Pawley refinement fit of PXRD data for **X-kdd-2-Cu**.

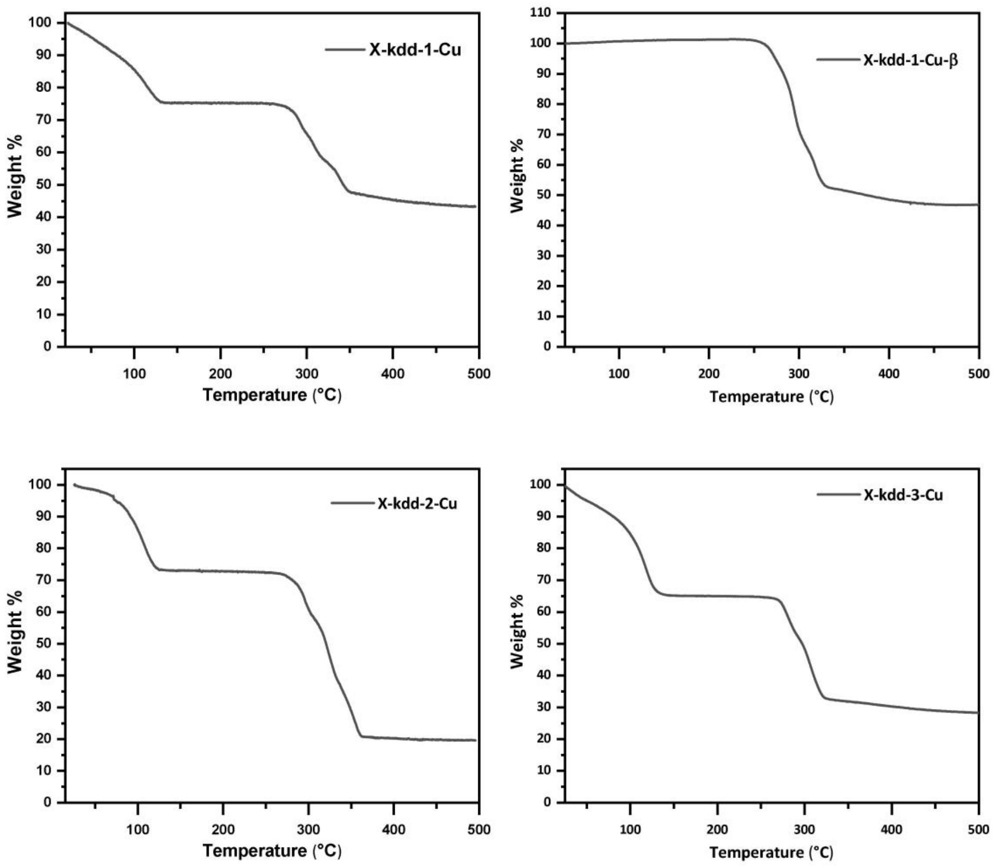

Figure S3. Thermogravimetric analysis (TGA) of as-synthesized samples of **X-kkd-1-Cu**, **X-kkd-2-Cu**, **X-kkd-3-Cu** and an activated sample of **X-kkd-1-Cu-β**.

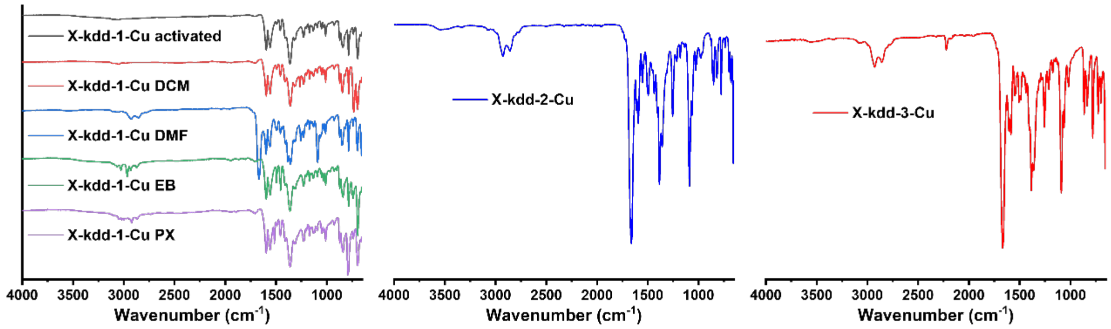

Figure S4. Fourier Transform Infrared (FTIR) spectra of **X-kdd-1-Cu-β**, **X-kdd-1-Cu-α$_{DCM}$**, **X-kdd-1-Cu-α$_{DMF}$**, **X-kdd-1-Cu-α$_{EB}$**, **X-kdd-1-Cu-α$_{PX}$**, **X-kdd-2-Cu** (as-synthesized) and **X-kdd-3-Cu** (as-synthesized).

X-kdd-1-Cu-β, v_{max} (cm^{-1}) = 1594, 1555, 1362, 1227, 1171, 1132, 1011, 840, 779, 688.

X-kdd-1-Cu-α$_{DCM}$, v_{max} (cm^{-1}) = 3049, 1600, 1555, 1357, 1265, 1232, 1116, 1127, 1011, 845, 779, 732, 696.

X-kdd-1-Cu-α$_{DMF}$, v_{max} (cm^{-1}) = 2932, 2847, 1667, 1597, 1553, 1357, 1251, 1227, 1089, 845, 779, 690, 660.

X-kdd-1-Cu-α$_{EB}$, v_{max} (cm^{-1}) = 3032, 2969, 2930, 2874, 1591, 1553, 1500, 1465, 1362, 1232, 1171, 1130, 1086, 1008, 837, 784, 693.

X-kdd-1-Cu-α$_{PX}$, v_{max} (cm^{-1}) = 3046, 3018, 2927, 2861, 1591, 1555, 1514, 1456, 1362, 1224, 1169, 1125, 1005, 848, 792, 688.

X-kdd-2-Cu, v_{max} (cm^{-1}) = 2924, 2855, 1661, 1591, 1547, 1495, 1384, 1246, 1086, 853, 770, 652.

X-kdd-3-Cu, v_{max} (cm^{-1}) = 2925, 2858, 2222, 1663, 1578, 1382, 1249, 1089, 864, 773, 654.

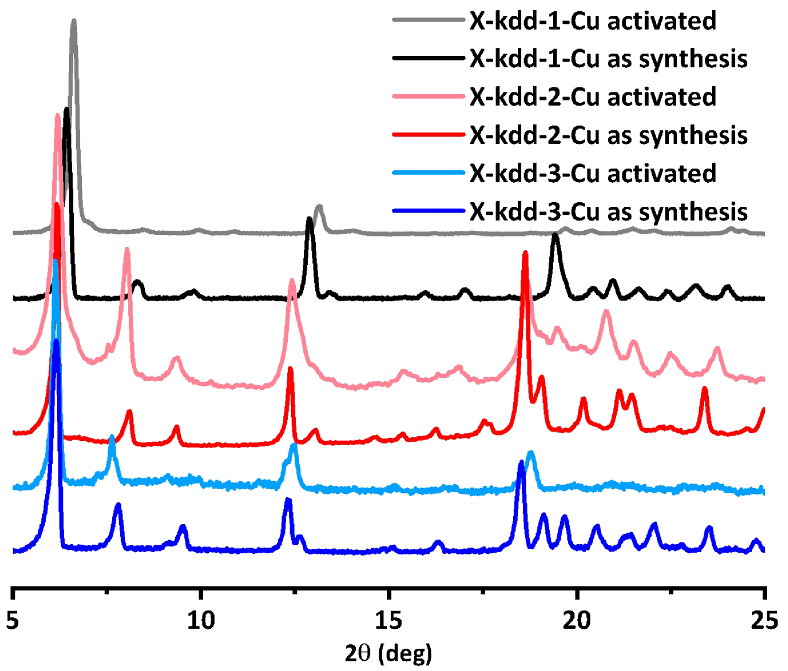

Figure S5. PXRD patterns of the as synthesized and activated phases of **X-kkd-1-Cu**, **X-kkd-2-Cu**, **X-kkd-3-Cu**.

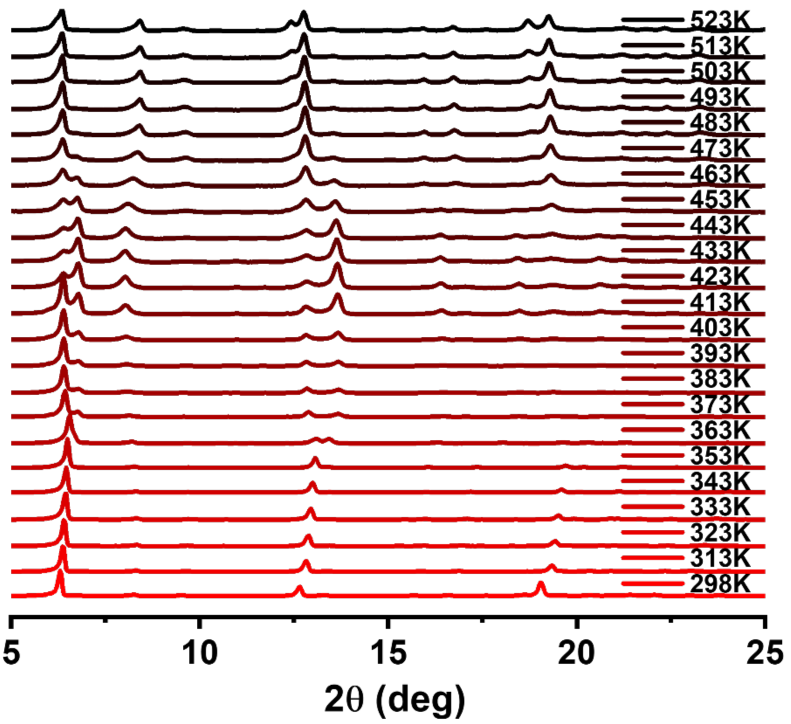

Figure S6. VT-PXRD patterns of **X-kkd-1-Cu** (conducted from an as-synthesized sample of **X-kdd-1-Cu-α$_{DMF}$** under N_2 flow).

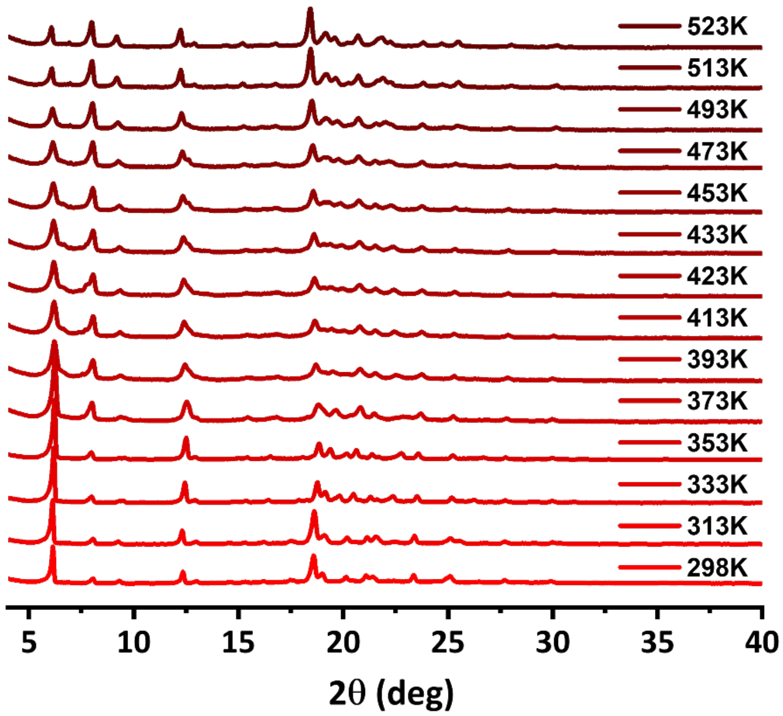

Figure S7. VT-PXRD patterns of **X-kkd-2-Cu** (conducted from an as-synthesized sample of **X-kdd-2-Cu** under N_2 flow).

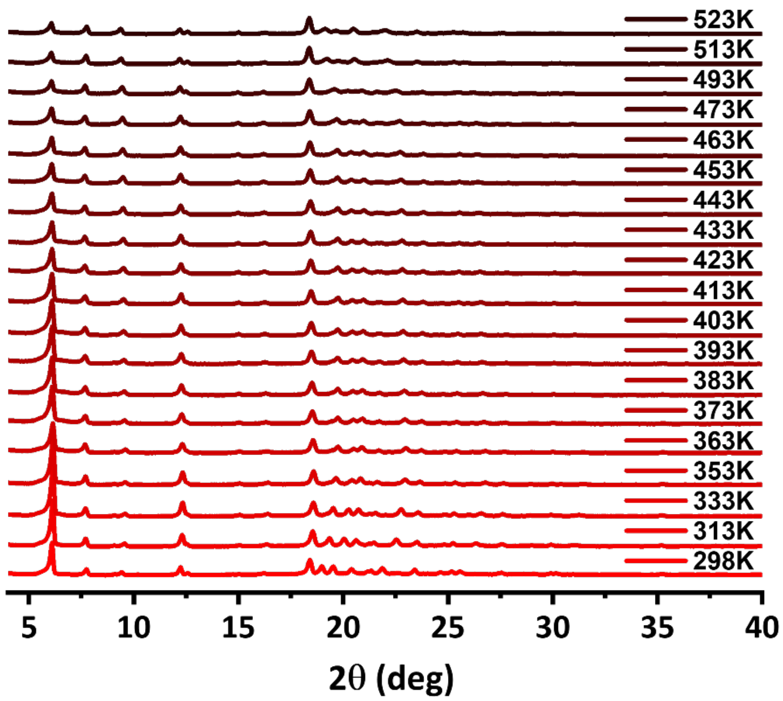

Figure S8. VT-PXRD patterns of **X-kkd-3-Cu** (conducted from an as-synthesized sample of **X-kdd-3-Cu** under N_2 flow).

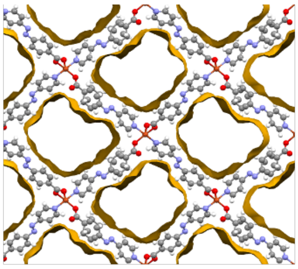

X-kdd-1-Cu-α
Voids: 46.4%

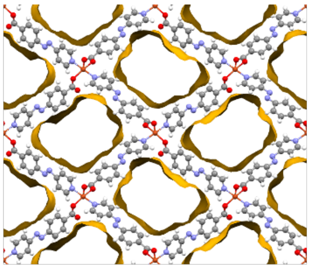

X-kdd-1-Cu-β
Voids: 43.7%

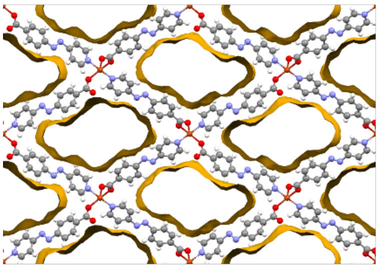

X-kdd-1-Cu-α_{DCM}
Voids: 43.4%

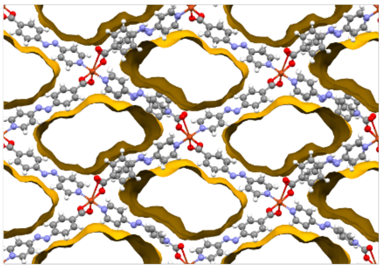

X-kdd-1-Cu-α_{PX}
Voids: 46.4%

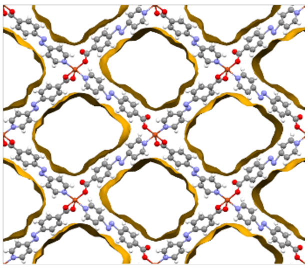

X-kdd-1-Cu-α_{EB}
Voids: 49.8%

Figure S9. View of the 3D frameworks and voids of **X-kdd-1-Cu-α_{DMF}**, **X-kdd-1-Cu-β**, **X-kdd-1-Cu-α_{DCM}**, **X-kdd-1-Cu-α_{PX}**, **X-kdd-1-Cu-α_{EB}**.

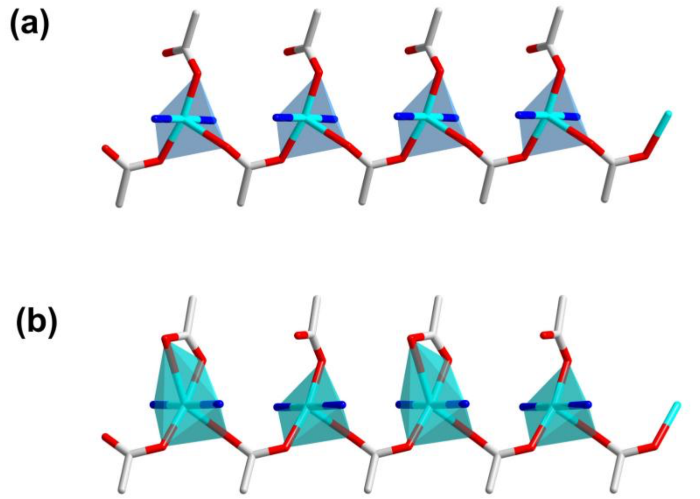

Figure S10. (a) Cu RBBs in **X-kdd-1-Cu-α$_{DMF}$, X-kdd-1-Cu-β, X-kdd-1-Cu-α$_{DCM}$** and **X-kdd-1-Cu-α$_{EB}$** and (b) **X-kdd-1-Cu-α$_{PX}$**.

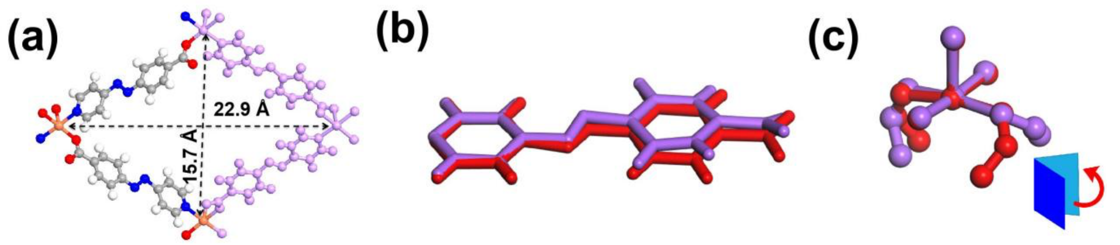

Figure S11. (a) Channel of **X-kdd-1-Cu-α_{DCM}**, Deformation of (b) ligand **1** and Cu coordination environment in **X-kdd-1-Cu-α_{DCM}.**

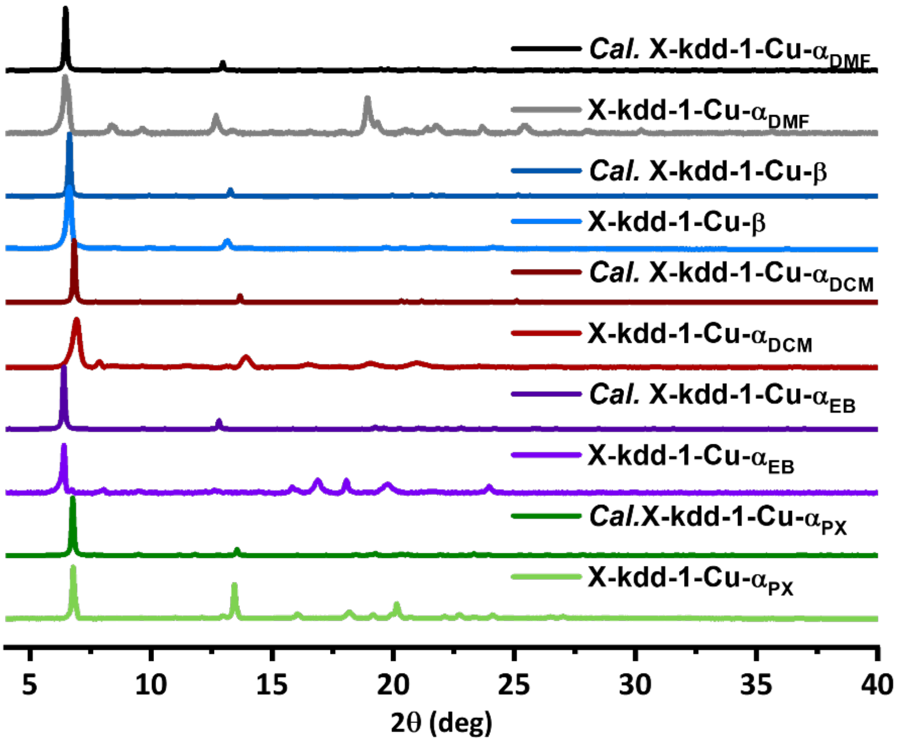

Figure S12. Experimental PXRD patterns of **X-kdd-1-Cu** under various conditions (as synthesized phase without further treatment **X-kdd-1-Cu-α_{DMF}**, activated phase **X-kdd-1-Cu-β**, DCM exchanged phase **X-kdd-1-Cu-α_{DCM}**, EB exchanged phase **X-kdd-1-Cu-α_{EB}**, PX exchanged phase **X-kdd-1-Cu-α_{PX}**) and comparison with PXRD patterns calculated from respective SCXRD determined structures.

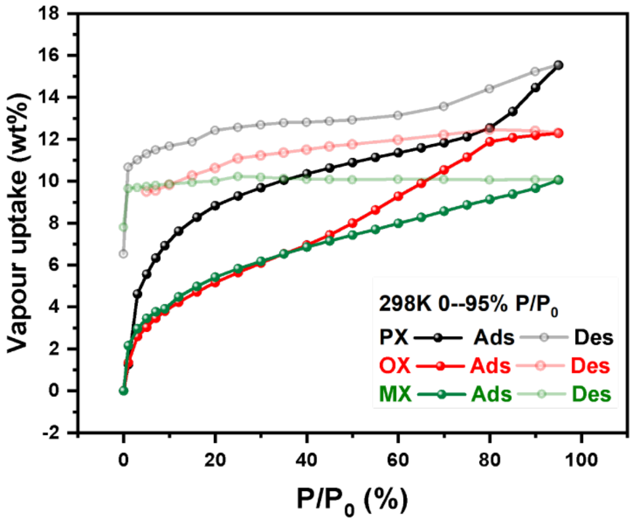

Figure S13. Vapor sorption isotherms of PX, MX and OX collected at 298 K from P/P_0 = 0 - 95% relative pressure on **X-kdd-1-Cu**.

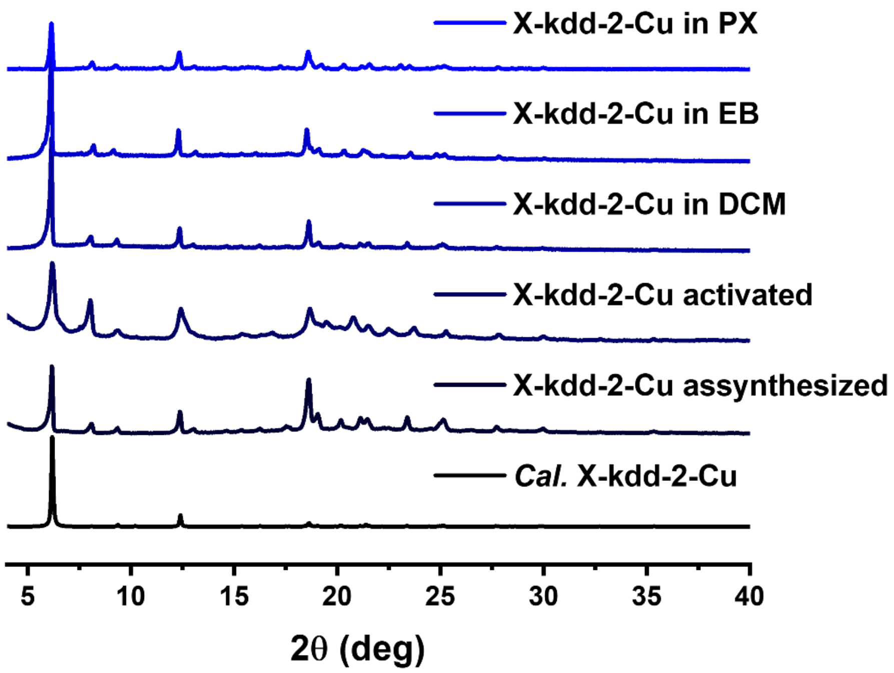

Figure S14. Experimental PXRD patterns of **X-kdd-2-Cu** under various conditions (as-synthesized sample without further treatment, activated sample, DCM exchanged sample, EB exchanged sample, PX exchanged sample) and comparison with PXRD pattern calculated from the simulated structure of **X-kdd-2-Cu**.

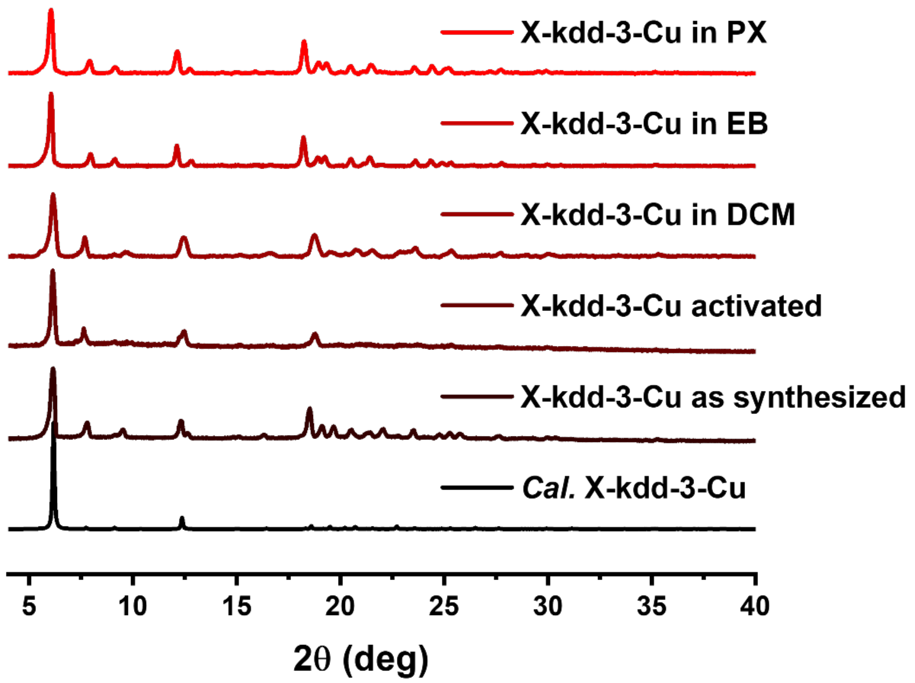

Figure S15. Experimental PXRD patterns of **X-kdd-3-Cu** under various conditions (as-synthesized sample without further treatment, activated sample, DCM exchanged sample, EB exchanged sample, PX exchanged sample) and comparison with PXRD pattern calculated from SCXRD determined structure of **X-kdd-3-Cu**.

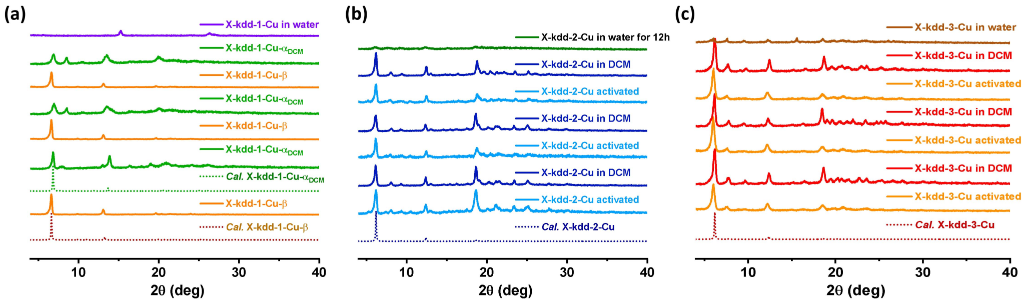

Figure S16. PXRD patterns reveal (a) reversible transformation between **X-kdd-1-Cu-α$_{\mathbf{DCM}}$** and **X-kdd-1-Cu-β** while (b) **X-kdd-2-Cu** and (c) **X-kdd-3-Cu** retained their structures upon DCM soaking/activation for 3 cycles. PXRD patterns of **X-kdd-1-Cu**, **X-kdd-2-Cu**, and **X-kdd-3-Cu** after soaking in water for 12 h indicate instability to water.

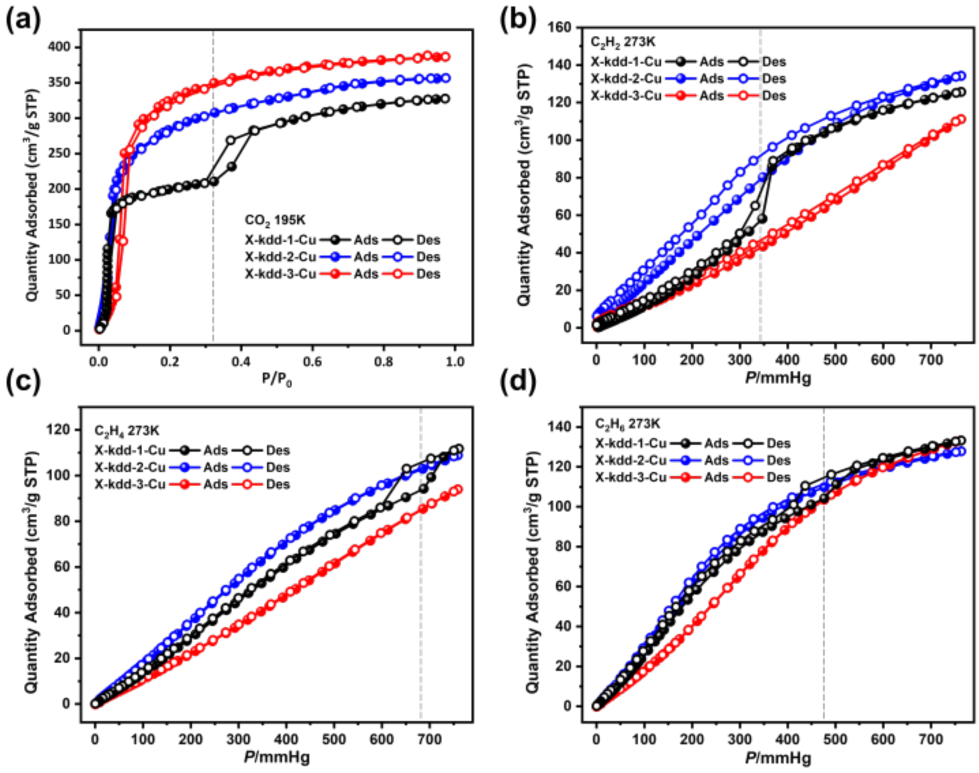

Figure S17. (a) CO_2 sorption isotherms of **X-kdd-1-Cu** (black), **X-kdd-2-Cu** (blue) and **X-kdd-3-Cu** (red) at 195K. (b) C_2H_2, (c) C_2H_4 and (d) C_2H_6 sorption isotherms of **X-kdd-1-Cu** (black), **X-kdd-2-Cu** (blue) and **X-kdd-3-Cu** (red) at 273K. Adsorption and desorption branches are indicated by solid and open symbols, respectively.

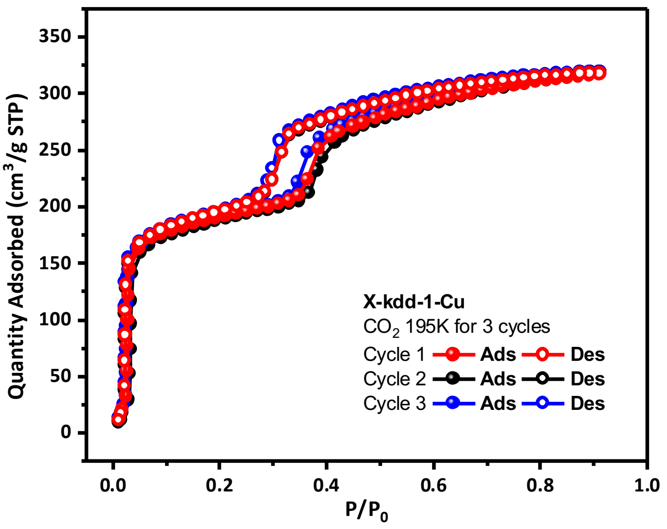

Figure S18. CO_2 sorption isotherms collected at 195 K on the same sample of **X-kdd-1-Cu** after three cycles of activation.

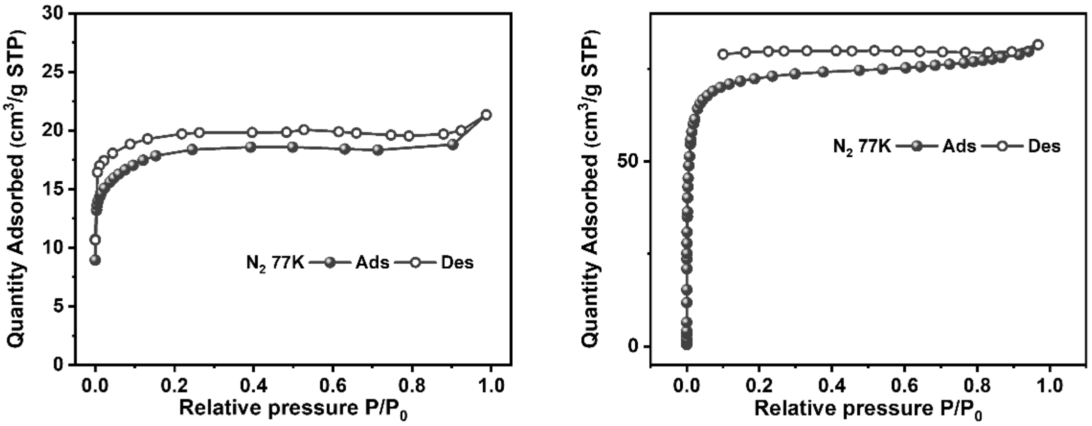

Figure S19. N_2 sorption isotherms of **kdd-1-Cu** (left) and **X-kdd-2-Cu** (right) at 77K, 0-1 bar.

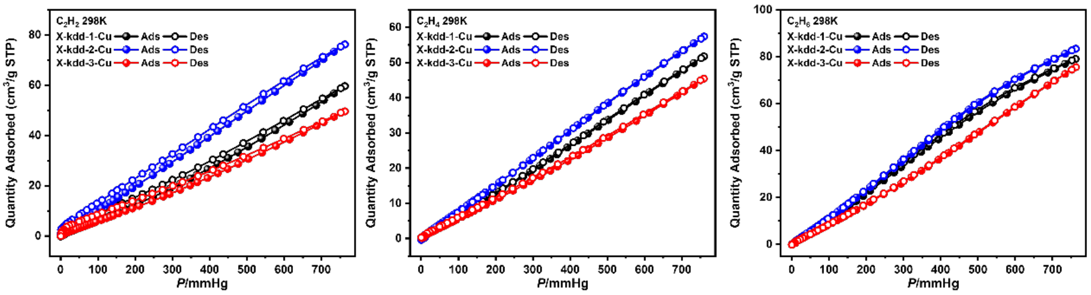

Figure S20. C_2H_2 (left), C_2H_4 (middle) and C_2H_6 (right) sorption isotherms of **X-kdd-1-Cu** (black), **X-kdd-2-Cu** (blue) and **X-kdd-3-Cu** (red) at 273K. Adsorption and desorption branches are indicated by solid and open symbols, respectively.

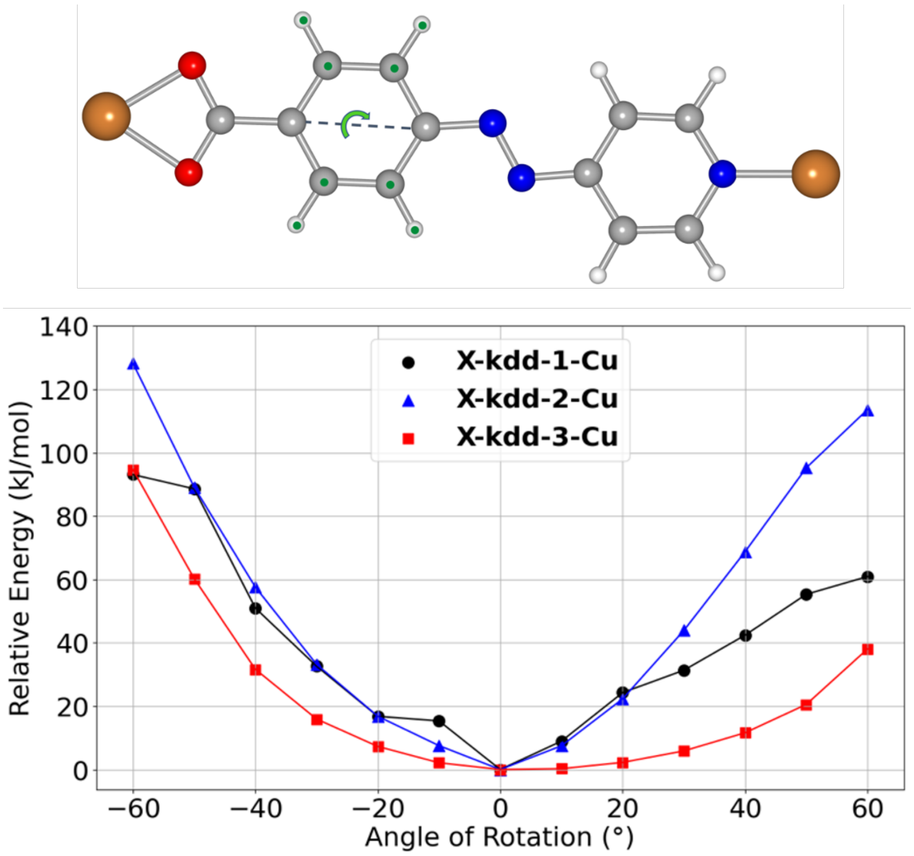

Figure S21. *Top*: Axis of rotation for half linker deformation in as-synthesized phase. The green dots mark the atoms that are rotated. Orange = Cu, Red = O, Grey = C, White = H, and Blue = N. *Bottom*: Potential energy curve of linkers **1**-**3** for rotations from –60 to +60 degrees from their preferred positions.

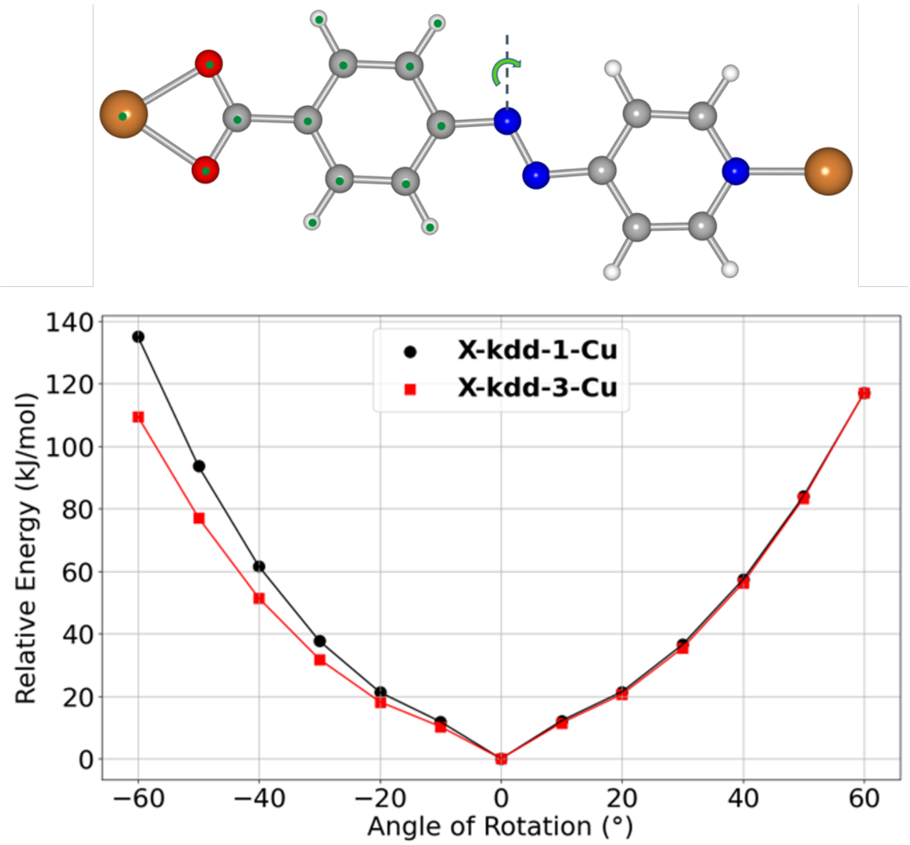

Figure S22. *Top*: Axis of rotation for half linker deformation in as-synthesized phase. The green dots mark the atoms that are rotated. Orange = Cu, Red = O, Grey = C, White = H, and Blue = N. *Bottom*: Potential energy curve of **X-kdd-1-Cu** and **X-kdd-3-Cu** linkers for deformations from –60 to +60 degrees.

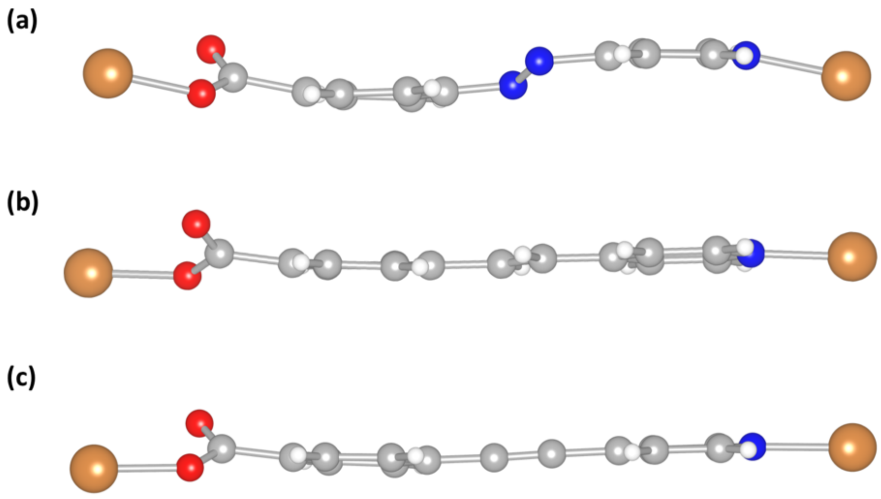

Figure S23. Side view of the three linkers from the relaxed structures. For clarity only the linker is shown, but the fully periodic structure was modeled. (a) **X-kdd-1-Cu**, (b) **X-kdd-2-Cu** and (c) **X-kdd-3-Cu**. Orange = Cu, Red = O, Grey = C, White = H, and Blue = N.

Table S1. CSD search results of 3D Cu RBB (Version 5.44, April 2023).

No.	Refcode	Dimension	No.	Refcode	Dimension
**1**	BACMOH	3D	**47**	HEDGUS	3D
**2**	BACMOH10	3D	**48**	HUTGIP	3D
**3**	BAHGUN	3D	**49**	HUTHEM	3D
**4**	BAHGUN01	3D	**50**	HUVGAJ	3D
**5**	BAHGUN02	3D	**51**	HUVKOB	3D
**6**	BAHGUN03	3D	**52**	LAYWEQ	3D
**7**	BAHGUN04	3D	**53**	MURRAT	3D
**8**	BUWGAC	3D	**54**	NELWIL	3D
**9**	BUWGAC01	3D	**55**	OPOJOU	3D
**10**	BUWGAC02	3D	**56**	PEFSOK	3D
**11**	BUWGAC03	3D	**57**	PEFTAX	3D
**12**	BUWGAC04	3D	**58**	QERZUI	3D
**13**	BUWGAC05	3D	**59**	QERZUI01	3D
**14**	BUWGAC06	3D	**60**	UFUMUD	3D
**15**	BUWGAC07	3D	**61**	UFUMUD01	3D
**16**	BUWGAC08	3D	**62**	UFUMUD02	3D
**17**	BUWGAC09	3D	**63**	UFUNAK	3D
**18**	BUWGAC10	3D	**64**	VEHPUU	3D
**19**	BUWGAC11	3D	**65**	VIGYUH	3D
**20**	BUWGAC12	3D	**66**	VOJPOA	3D
**21**	BUWGAC13	3D	**67**	VOJPUG	3D
**22**	BUWGAC14	3D	**68**	VOJPUG01	3D
**23**	BUWGAC15	3D	**69**	VURYIR	3D
**24**	BUWGAC16	3D	**70**	VURYOX	3D
**25**	BUWGAC17	3D	**71**	WAHFUJ	3D
**26**	BUWGAC18	3D	**72**	WAHFUJ01	3D
**27**	BUWGAC19	3D	**73**	WAHGAQ	3D
**28**	BUWGAC20	3D	**74**	WASRAK	3D
**29**	BUWGAC21	3D	**75**	UFUNIS	3D
**30**	BUWGAC22	3D	**76**	AQUBIY	2D
**31**	BUWGAC23	3D	**77**	ATODES	2D
**32**	BUWGAC24	3D	**78**	BEQHOW	2D
**33**	BUWGEG	3D	**79**	BEXDUE	2D
**34**	CELJUA	3D	**80**	BUGJAO	2D
**35**	CELKAH	3D	**81**	DUMMAB	2D
**36**	CELKEL	3D	**82**	JERLIC	2D
**37**	EJOGOZ	3D	**83**	JEXPAE	2D
**38**	FEFCAY	3D	**84**	KAYZER	2D
**39**	FEVNOK	3D	**85**	KEPSAA	2D
**40**	FEXHOG	3D	**86**	KEPSEE	2D

**41**	FEXHOG01	3D	**87**	MIWJIM	2D
**42**	GIXMOR	3D	**88**	MIWJUY	2D
**43**	GIXMUX	3D	**89**	ODEYIG	2D
**44**	GLALCU10	3D	**90**	OHIRED	2D
**45**	GMETCU	3D	**91**	OHIRED01	2D
**46**	HAJJAE	3D	**92**	OHIRED02	2D
**93**	OKEKUN	2D			
**94**	OMEMOL	2D			
**95**	OVAZOB	2D			
**96**	PERZOC	2D			
**97**	PEZRES	2D			
**98**	SABWAV	2D			
**99**	SABWID	2D			
**100**	TISCAC	2D			
**101**	VUVQOU	2D			

Table S2. Crystallographic data and structure refinement summary for **X-kdd-1-Cu**.

**Compounds**	**X-kdd-1-Cu-α$_{DMF}$**	**X-kdd-1-Cu-β**	**X-kdd-1-Cu-α$_{PX}$**
Identification code	2244648	2244646	2244647
Empirical formula	$C_{24}H_{16}CuN_6O_4$	$C_{24}H_{16}CuN_6O_4$	$C_{160}H_{144}Cu_4N_{24}O_{16}$
Formula weight	515.97	515.97	2915.16
Temperature/K	100	100	100
Crystal system	monoclinic	monoclinic	triclinic
Space group	*Cc*	*Cc*	*P1*
a/Å	4.8958(2)	4.7470(4)	9.8141(4)
b/Å	41.6463(17)	41.534(4)	15.9564(6)
c/Å	18.0904(8)	17.4245(16)	23.2243(9)
α/°	90	90	89.201(2)
β/°	90.5900(10)	93.919(2)	83.930(2)
γ/°	90	90	82.899(2)
Volume/Å^3	3688.3(3)	3427.4(5)	3588.7(2)
Z	4	4	1
Pcalc g/cm^3	0.929	1	1.349
μ/mm^{-1}	0.62	0.667	1.264
F(000)	1052	1052	1518
Radiation	MoKα (λ = 0.71073)	MoKα (λ = 0.71073)	CuKα (λ = 1.54178)
2Θ range for data collection/°	5.966 to 55.13	5.08 to 55.2	5.582 to 140.456
Index ranges	-6 ≤ h ≤ 6, -54 ≤ k ≤ 54, -23 ≤ l ≤ 23	-6 ≤ h ≤ 6, -54 ≤ k ≤ 54, -22 ≤ l ≤ 22	-11 ≤ h ≤ 11, -18 ≤ k ≤ 19, -26 ≤ l ≤ 28
Reflections collected	45259	58759	41627
Independent reflections	8503 [R_{int} = 0.0482, R_{sigma} = 0.0441]	7897 [R_{int} = 0.1619, R_{sigma} = 0.1317]	17560 [R_{int} = 0.0643, R_{sigma} = 0.0719]
Data/restraints/parameters	8503/26/353	7897/89/305	17560/93/1854
Goodness-of-fit on F^2	1.041	1.012	1.047
Final R indexes [I>=2σ (I)]	R1^a = 0.0388, wR2^b = 0.0971	R1^a = 0.1027, wR2^b = 0.2375	R1^a = 0.0834, wR2^b = 0.2246
Final R indexes [all data]	R1^a = 0.0548, wR2^b = 0.1029	R1^a = 0.1880, wR2^b = 0.2861	R1^a = 0.1132, wR2^b = 0.2527
Largest diff. peak/hole / e Å^{-3}	0.56/-0.34	1.05/-0.51	0.81/-0.88
Flack parameter	0.342(14)	0.40(5)	0.34(5)

$^aR_1=\Sigma||F_o|-|F_c||/\Sigma|F_o|$. $^bwR_2= |\Sigma w(|F_o|^2-|F_c|^2)|/\Sigma|w(Fo^2)^2|^{1/2}$

Compounds	X-kdd-1-Cu-α_{DCM}	X-kdd-1-Cu-α_{EB}
Identification code	2244645	2244644
Empirical formula	$C_{24}H_{16}CuN_6O_4$	$C_{24}H_{16}CuN_6O_4$
Formula weight	515.97	515.97
Temperature/K	100	110
Crystal system	monoclinic	monoclinic
Space group	*Cc*	*Cc*
a/Å	4.7174(6)	4.8721(2)
b/Å	45.848(5)	42.4868(14)
c/Å	15.691(2)	18.2266(7)
α/°	90	90
β/°	91.788(3)	93.537(3)
γ/°	90	90
Volume/Å^3	3391.9(7)	3765.7(2)
Z	4	4
ρcalc g/cm^3	1.01	0.91
μ/mm^{-1}	0.674	1.051
F(000)	1052	1052
Radiation	MoKα (λ = 0.71073)	CuKα (λ = 1.54178)
2Θ range for data collection/°	5.194 to 55.056	4.16 to 140.388
Index ranges	-6 ≤ h ≤ 6, -58 ≤ k ≤ 57, -20 ≤ l ≤ 20	-5 ≤ h ≤ 4, -49 ≤ k ≤ 51, -22 ≤ l ≤ 22
Reflections collected	36359	18082
Independent reflections	7681 [R_{int} = 0.1128, R_{sigma} = 0.1186]	5861 [R_{int} = 0.0969, R_{sigma} = 0.0965]
Data/restraints/parameters	7681/92/317	5861/105/317
Goodness-of-fit on F^2	0.986	1.024
Final R indexes [I>=2σ (I)]	R1^a = 0.0707, wR2^b = 0.1592	R1^a = 0.0856, wR2^b = 0.2190
Final R indexes [all data]	R1^a = 0.1213, wR2^b = 0.1810	R1^a = 0.1276, wR2^b = 0.2559
Largest diff. peak/hole / e Å^{-3}	0.71/-0.66	0.44/-0.61
Flack parameter	0.46(2)	0.49(8)

$^aR_1=\Sigma||F_o|-|F_c||/\Sigma|F_o|$. $^bwR_2= [\Sigma w(|F_o|^2-|F_c|^2)]/\Sigma|w(Fo^2)^2|^{1/2}$

Table S3. The pattern matching analysis data for **X-kdd-2-Cu** by the Pawley refinement.

	**X-kdd-2-Cu**	
Indexed lattice parameters	a = 5.10 Å	α = 90˚
	b = 43.54 Å	β = 95.65˚
	c = 18.87 Å	γ = 90˚
Refined lattice parameters	a = 5.12 Å	α = 90˚
	b = 43.61 Å	β = 95.77˚
	c = 18.96 Å	γ = 90˚
R_p	4.30%	
R_{wp}	8.51%	

Table S4. Comparative analysis of the structural differences between the three isolated phases (α_{DMF}, β and α_{PX}) of **X-kdd-1-Cu**.

		**X-kdd-1-Cu-α_{DMF}**	**X-kdd-1-Cu-β**	**X-kdd-1-Cu-α_{PX}**[a]
Bond length and bond angle 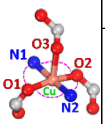	Cu-O1 (Å)	1.972(3),	1.952(8),	1.954(7),1.953(7),1.967(7),1.949(7),
	Cu-O2 (Å)	1.941(4),	1.988(11),	1.944(7),1.960(7),1.749(7),1.957(7),
	Cu-O3 (Å)	2.303(3),	2.202(12),	2.306(7),2.330(7),2.338(8),2.291(7),
	Cu-N1 (Å)	2.023(4),	1.984(18),	2.040(8),2.030(8),2.034(8),2.041(8),
	Cu-N2 (Å)	2.021(4)	2.069(15)	2.019(9),2.012(9),2.007(9),2.030(9)
	∠O1-Cu-N1 (°)	91.7(2),	92.5(4),	89.0(3),91.1(3),88.9(3),93.4(3),
	∠O2-Cu-N1 (°)	86.6(1),	86.5(4),	93.4(3),88.9(3),91.4(30),89.1(3),
	∠O2-Cu-N2 (°)	93.8(2),	93.6(6),	83.1(3),94.8(3),85.1(3),95.2(3),
	∠O1-Cu-N2 (°)	85.5(1)	88.3(4)	95.1(3),85.4(3),94.8(3),83.0(3)
Dihedral angles between carboxyl plane and phenyl plane of ligand **1**.[b] 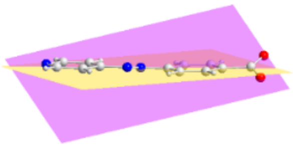		20.86(28)°,<br>8.64(71)°,<br>32.19(15)°	28.54(10)°,<br>6.17(10)°	19.60(63)°,<br>30.06(76)°,<br>23.86(84)°,<br>19.24(62)°,<br>24.32(85)°,<br>20.93(68)°,<br>29.21(74)°,<br>20.24(61)°
Dihedral angles between pyridyl plane and phenyl plane of ligand **1**.[b] 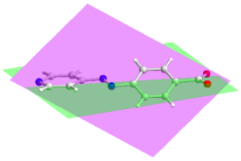		3.31(19)°,<br>12.94(57)°,<br>27.97(16)°	5.76(47)°,<br>4.98(51)°	2.24(37)°,<br>31.22(54)°,<br>29.69(61)°,<br>2.15(48)°,<br>30.13(57)°,<br>1.38(52)°,<br>29.74(53)°,<br>1.828(50)°
Edge lengths, diagonal distances and angles of quadrangular channel pores along *a* axis 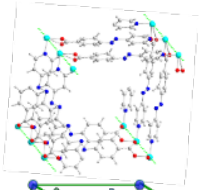 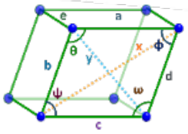	a (Å)	13.369(1),	13.119(3),	14.347(1),
	b (Å)	14.225(1),	13.119(3),	13.622(2),
	c (Å)	14.225(1),	13.973(3),	13.628(1),
	d (Å)	13.369(1),	13.973(3),	14.395(1),
	e (Å)	4.8958(5),	4.909(9),	4.903(1),
	x (Å)	20.823(1),	17.384(3),	23.101(1),
	y (Å)	18.089(1),	20.767(3),	15.801(1),
	θ (°)	97.941(3),	97.015(14),	110.965(4),
	ψ (°)	78.966(1),	79.960(19),	71.018(4),
	ω (°)	97.941(3),	103.066(24),	111.451(2),
	ϕ (°)	85.152(5).	79.960(17).	66.566(2).

View along *a* axis of **X-kdd-1-Cu's kdd** nets structure.	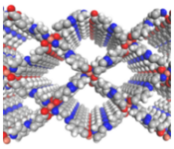	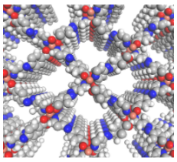	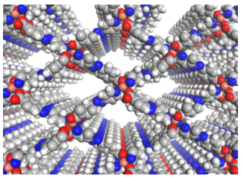

[a]*Multiple values come from low symmetric of* ***X-kdd-1-Cu-*** $\boldsymbol{\alpha}_{PX}$*,*

[b]*Multiple values come from ligand disorder in* ***X-kdd-1-Cu-*** $\boldsymbol{\alpha}_{DMF}$*.*

Table S5. Saturated gas uptake of **X-kdd-1-Cu**, **X-kdd-2-Cu** and **X-kdd-3-Cu**.

	**X-kdd-1-Cu**	**X-kdd-2-Cu**	**X-kdd-3-Cu**
CO_2 at 195 K, P/P_0 = 1	324	330	375
N_2 at 77 K, P/P_0 = 1	21	81	30
Ar at 87 K, P/P_0 = 1	--	--	360

Table S6. Cell parameters of **X-kdd-3-Cu (published as-synthesized)**, **X-kdd-3-Cu (as-synthesized)**, **X-kdd-3-Cu (PX)** structures.

	X-kdd-3-Cu (previously published, as-synthesized)	X-kdd-3-Cu (as-synthesized)	X-kdd-3-Cu (PX)
Identification code	2145277	2447687	2447686
a (Å)	4.8634	4.7427(3)	4.8281(4)
b (Å)	45.644	46.285(3)	45.517(2)
c (Å)	18.3918	17.552(2)	18.2241(12)
α (º)	90	90	90
β (º)	93.659	91.110(8)	92.156(7)
γ (º)	90	90	90
V ($Å^3$)	4074.38	3852.3(5)	4002.1(5)
Ligand **3** in X-kdd-3-Cu (previously published, as-synthesized)	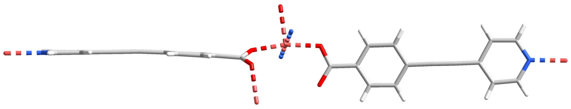		
Ligand **3** in X-kdd-3-Cu (as-synthesized)	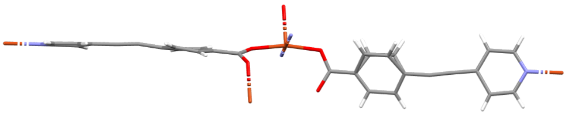		
Ligand **3** in X-kdd-3-Cu (PX)	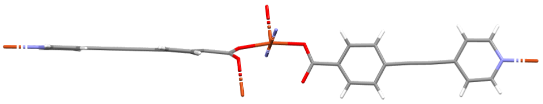		

Table S7. Calculated bulk moduli of **X-kdd-1-Cu** and **X-kdd-3-Cu** in kBar.

**X-kdd-1-Cu**	114.2
**X-kdd-3-Cu**	121.6

11. References

(1) Oldknow, S.; Martir, D. R.; Pritchard, V. E.; Blitz, M. A.; Fishwick, C. W. G.; Zysman-Colman, E.; Hardie, M. J. Structure-Switching M3L2 Ir(Iii) Coordination Cages with Photo-Isomerising Azo-Aromatic Linkers. *Chem. Sci.* **2018**, *9* (42), 8150–8159.

(2) Wang, X.; Wang, F.; Zhang, C.; Wang, Q. Regulating the Proton Conductivity of Metal Organic Framework Materials through Solvent Control. *New J. Chem.* **2022**, *46* (14), 6657–6662.

(3) Wang, S. M.; Mu, X. T.; Liu, H. R.; Zheng, S. T.; Yang, Q. Y. Pore-Structure Control in Metal–Organic Frameworks (MOFs) for Capture of the Greenhouse Gas SF6 with Record Separation. *Angew. Chem. Int. Ed.* **2022**, *61* (33).

(4) Wang, S. M.; Yang, Q. Y. A Copper-Based Metal-Organic Framework for Upgrading Natural Gas through the Recovery of C2H6 and C3H8. *Green Chem. Eng.* **2022**, No. March, 6–12.

(5) Sheldrick, G. M.; Bruker, A. X. S. Inc., Madison, WI, 2000;(b) GM Sheldrick. *Acta Crystallogr., Sect. A Fundam. Crystallogr* **2015**, *71*, 3–8.

(6) Krause, L.; Herbst-Irmer, R.; Sheldrick, G. M.; Stalke, D. Comparison of Silver and Molybdenum Microfocus X-Ray Sources for Single-Crystal Structure Determination. *J. Appl. Crystallogr.* **2015**, *48* (1), 3–10.

(7) Dolomanov, O. V.; Bourhis, L. J.; Gildea, R. J.; Howard, J. A. K.; Puschmann, H. OLEX2: A Complete Structure Solution, Refinement and Analysis Program. *J. Appl. Crystallogr.* **2009**, *42* (2), 339–341.

(8) Sheldrick, G. M. Crystal Structure Refinement with SHELXL. *Acta Crystallogr. Sect. C Struct. Chem.* **2015**, *71* (Md), 3–8.

(9) Kresse, G.; Furthmüller, J. Efficient Iterative Schemes for Ab Initio Total-Energy Calculations Using a Plane-Wave Basis Set. *Phys. Rev. B - Condens. Matter Mater. Phys.* **1996**, *54* (16), 11169–11186.

(10) Kresse, G.; Joubert, D. From Ultrasoft Pseudopotentials to the Projector Augmented-Wave Method. *Phys. Rev. B* **1999**, *59* (3), 1758–1775.

(11) Thonhauser, T.; Zuluaga, S.; Arter, C. A.; Berland, K.; Schröder, E.; Hyldgaard, P. Spin Signature of Nonlocal Correlation Binding in Metal-Organic Frameworks. *Phys. Rev. Lett.* **2015**, *115* (13), 136402.

(12) Thonhauser, T.; Cooper, V. R.; Li, S.; Puzder, A.; Hyldgaard, P.; Langreth, D. C. Van Der Waals Density Functional: Self-Consistent Potential and the Nature of the van Der Waals Bond. *Phys. Rev. B* **2007**, *76* (12), 125112.

(13) Berland, K.; Cooper, V. R.; Lee, K.; Schröder, E.; Thonhauser, T.; Hyldgaard, P.; Lundqvist, B. I. Van Der Waals Forces in Density Functional Theory: A Review of the VdW-DF Method. *Reports Prog. Phys.* **2015**, *78* (6), 66501.

(14) Langreth, D. C.; Lundqvist, B. I.; Chakarova-Käck, S. D.; Cooper, V. R.; Dion, M.; Hyldgaard, P.; Kelkkanen, A.; Kleis, J.; Kong, L.; Li, S. A Density Functional for Sparse Matter. *J. Phys. Condens. Matter* **2009**, *21* (8), 84203.

(15) Wang, L.; Maxisch, T.; Ceder, G. Oxidation Energies of Transition Metal Oxides within the GGA+ U Framework. *Phys. Rev. B* **2006**, *73* (19), 195107.
